# High Charge Density in Peptide Dendrimers is Required to Destabilize Membranes: Insights into Endosome Evasion

**DOI:** 10.1021/acs.jcim.4c00018

**Published:** 2024-04-08

**Authors:** Filipe E. P. Rodrigues, Tamis Darbre, Miguel Machuqueiro

**Affiliations:** † BioISI—Instituto de Biossistemas e Ciências Integrativas Faculdade de Ciências, Universidade de Lisboa, Lisboa 1749-016, Portugal; ‡ Department of Chemistry Biochemistry and Pharmaceutical Sciences, University of Bern, Bern 3012, Switzerland

## Abstract

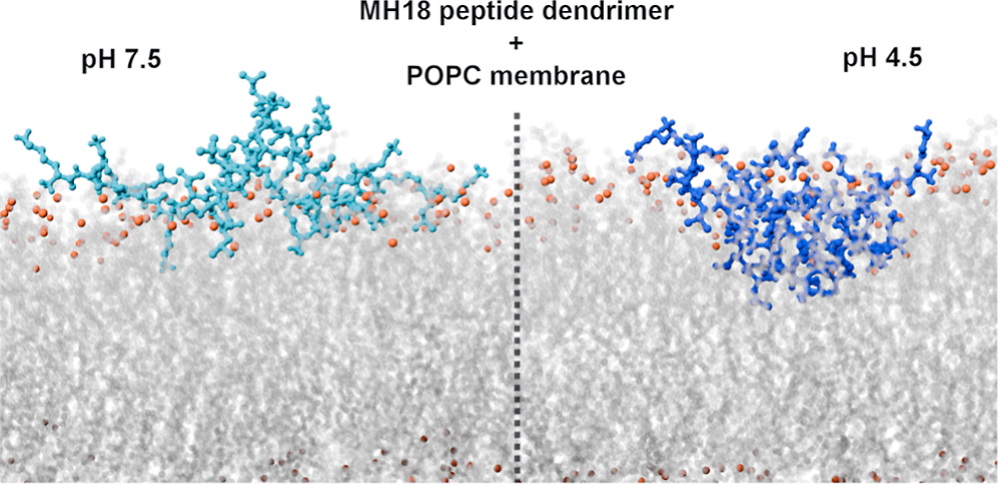

Peptide dendrimers are a type of branched, symmetric, and topologically well-defined molecule that have already been used as delivery systems for nucleic acid transfection. Several of the most promising sequences showed high efficiency in many key steps of transfection, namely, binding siRNA, entering cells, and evading the endosome. However, small changes to the peptide dendrimers, such as in the hydrophobic core, the amino acid chirality, or the total available charges, led to significantly different experimental results with unclear mechanistic insights. In this work, we built a computational model of several of those peptide dendrimers (MH18, MH13, and MH47) and some of their variants to study the molecular details of the structure and function of these molecules. We performed CpHMD simulations in the aqueous phase and in interaction with a lipid bilayer to assess how conformation and protonation are affected by pH in different environments. We found that while the different peptide dendrimer sequences lead to no substantial structural differences in the aqueous phase, the total charge and, more importantly, the total charge density are key for the capacity of the dendrimer to interact and destabilize the membrane. These dendrimers become highly charged when the pH changes from 7.5 to 4.5, and the presence of a high charge density, which is decreased for MH47 that has four fewer titratable lysines, is essential to trigger membrane destabilization. These findings are in excellent agreement with the experimental data and help us to understand the high efficiency of some dendrimers and why the dendrimer MH47 is unable to complete the transfection process. This evidence provides further understanding of the mode of action of these peptide dendrimers and will be pivotal for the future design of new sequences with improved transfection capabilities.

## 1 Introduction

Dendrimers are a family of tree-like molecules with a well-defined and homogeneous structure. These structures consist of a core that radially branches symmetrically, forming generations at each branching level. With an increased number of generations, their structure is thought to acquire a more globular shape.^1^ Even if the exact structure adopted by dendrimers in solution is still somewhat undefined, their topology limits the overall structural variability.^1^ They are quite versatile and adaptable molecules because of their inherent multivalency and the simplicity with which their properties can be changed by altering their constitution or grafting new functional groups.^1−3^

There are different types of dendrimers, which can be classified based on their constitution, such as poly(amidoamine) (PAMAM) dendrimers, poly(propyleneimine) (PPI) dendrimers, and peptide dendrimers. PAMAM dendrimers have a high surface area and depending on the number of generations, can reach a size comparable to a medium-sized protein.^4^ Another type of dendrimer, the PPIs, as well as PAMAM, are molecules with potential use for several applications in the chemical and biomedical fields and have been thoroughly explored in the literature.^3−5^ However, regardless of their important properties, most of these molecules have cytotoxicity problems, due to their large molecular size, the high surface charge, and their immunogenicity.^5^

Peptide dendrimers are characterized by being partially or fully constituted by amino acid residues. In addition to the properties already described for other types of dendrimers, these have the advantage of being less immunogenic.^6,7^ They have been reported to interact with several biological targets, leading to good activity as antimicrobial agents,^8^ pathogenic biofilm inhibitors,^8^ drug delivery systems,^9^ and superior vectors for siRNA and small oligonucleotides.^6,7^ Cationic peptide dendrimers have been shown to efficiently transfect DNA/RNA and protect them from degradation.^6,7^ The positive charges from lysine and arginine residues help the binding to negatively charged nucleic acids and favor membrane interactions, which are often slightly anionic.^10^ The adhesion to the cell membrane may also facilitate its entry via endosome-mediated internalization. However, the first transfecting peptide dendrimers required the addition of cationic lipids, such as lipofectin, to be active. More recently, peptide dendrimers with covalently attached lipophilic tails showed efficient transfection without lipofectin.^6,7,11,12^

**Figure 1:**
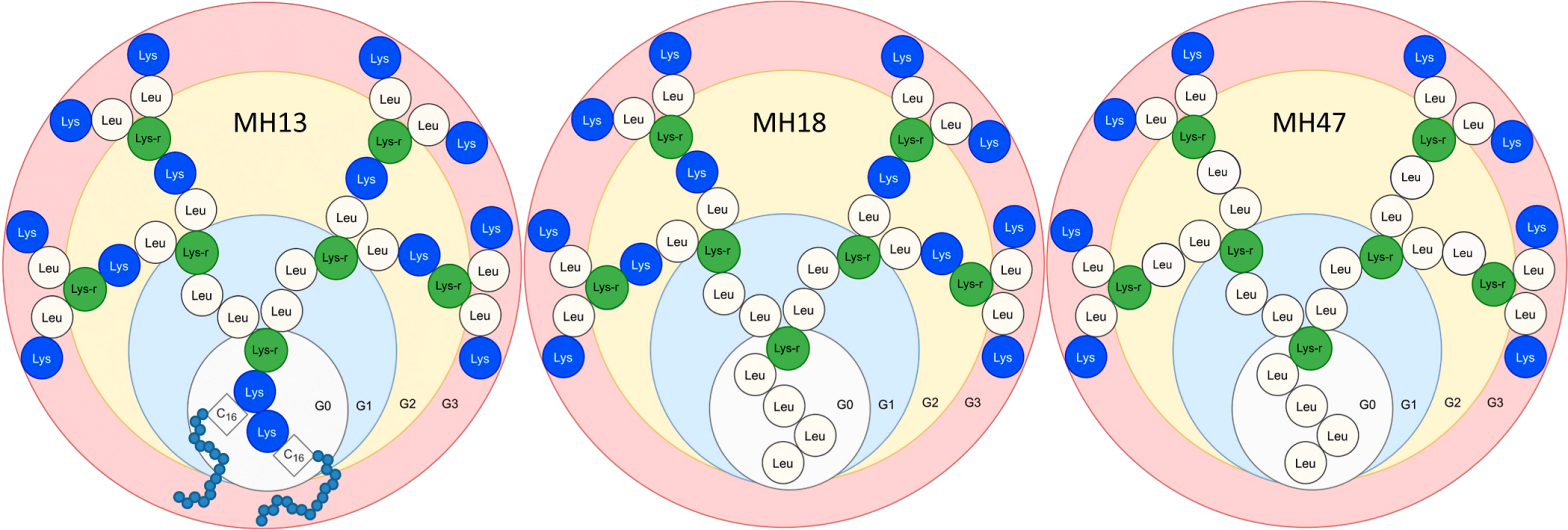
Schematic representation of peptide dendrimers MH13, MH18, and MH47. The different generations, G0, G1, G2, and G3 are identified and marked with different colored regions. All residues are depicted by a colored circle, with leucines in white, regular lysines in blue, and branching lysines in green. The two lysines from MH13 G0 are covalently bound to a palmitoyl group each and are depicted with 16 smaller gray circles.

Two very efficient third-generation peptide dendrimer sequences, MH18 and MH13 (Figure 1A,B),^10,11^ are constituted by leucines and lysines, with a tetra-leucine or two palmitoyl groups as a hydrophobic core, respectively. By changing the chirality of these sequences and performing a few mutations in the sequences, it was possible to modulate the affinity for nucleic acids and their performances as transfecting agents. Several sequences emerged from these efforts, like MH47 (Figure 1C), which has four lysines with free side chains in G2 mutated to leucines. This dendrimer tightly binds nucleic acids, but acts as a poor transfection agent. Additionally, peptide dendrimers with variations in the chirality of the MH13 and MH18 amino acids, where some or all residues were replaced by their d-enantiomers, gave different results in RNA transfection.^7^

Despite the experimental findings on these peptide dendrimers, there are still important questions at the molecular level that need to be answered to help unveil the relationship between their structure and efficiencies. For instance, it is not well-understood why changing different combinations of residues from MH18 from l to d-amino acids leads to different effects in the affinity to RNA, transfection efficiency, and release of nucleic acids.^7^ Similarly, replacing lysines for other potentially cationic residues, namely histidines also leads to a decreased efficacy.^6^ Furthermore, while it is accepted that cationic peptide dendrimers internalize the cell via the endosome and the endosomal escape is facilitated by the acidification of the late endosome, the molecular mechanism of this process is still unclear.

Computational methods have proven to be invaluable in studying these processes since they allow us to peer into molecular details that are hard to attain, if even possible, through most experimental methods. Due to their flexible nature, it is difficult to obtain clear structural data of the dendrimers in solution using experimental techniques. This has been mitigated using computational methods, such as the use of molecular dynamics (MD) simulations^7,13−16^ or Constant-pH MD (CpHMD) simulations,^17,18^ which take into account the pH effects on the dendrimer conformational spaces. These state-of-the-art methodologies are still currently being developed to address known limitations related to the size of the solutes, the number of titrable residues, and the inhomogeneity of the media where the electrostatic interactions occur.^19−23^ By uncovering the molecular mechanisms of dendrimers and their interactions with different biomolecules, we can propose sequence modifications aimed at enhancing their efficacy.

In this work, we present a structural characterization in water of MH13, MH18, MH47, and some of their variants as well as the effect of pH on their structure. We also present a more in-depth study of MH18, MH18D3, MH13, and MH47 interacting with a POPC membrane model, which elucidates some of its pH-dependent mechanisms regarding its interaction with biological membranes.

## 2 Methods

### 2.1 Dendrimer Setup

We used PyMOL^24^ to build the initial conformations of the dendrimers. The free lysine side chains, as well as the free N-termini, are titrated, while the C-terminus is capped with NH_2_ groups. For both the ramified and palmitoylated lysines, we built additional blocks on our force field. In the ramified lysine, the amine group is changed for an amide bond, with identical parameters to the ones used in the main chain. For the palmitoylated lysine, used as a hydrophobic core of some peptide dendrimer sequences, we adapted the palmitoyl parameters from 1-palmitoyl-2-oleoyl-glycero-3-phosphocholine (POPC) lipids and coupled them to a ramified lysine, previously described.

For the membrane simulations, a lipid bilayer of 304 POPC molecules was prepared. To build this membrane patch, we started with a pre-equilibrated bilayer with 128 POPC lipids^25^ and multiplied it by 4 in the *x*/*y* plane using the GROMACS tool genconf, obtaining a membrane patch of 512 lipids. From this, we applied a shrinking procedure where 8 lipids, 4 from each monolayer, are randomly removed in a stepwise manner until we reached our target number of 304 lipids.^25^ In each step, we relaxed our system using a short MD segment (∼1 ns) and using a stronger pressure (50 bar) in the *x*/*y* plane to induce rapid convergence of the total area. In all of these steps, water molecules can also be randomly removed to satisfy the water/lipid ratio requested. We prepared our systems with a very high ratio of 78:1 to preserve all the initial water molecules since we wanted to create a box large enough in height to allow the dendrimer to be placed far away from the membrane in the starting configurations.^26^ After the final membrane patch of 304 lipids was obtained, the membrane was then equilibrated at normal pressure.

The dendrimers were prepared in a fully protonated form, placed ∼3 nm away from the membrane, and simulated using unbiased MD to sample their unbiased membrane approach pathways. The simulations were stopped when the dendrimer reached the nonbonded interactions cutoff distance (∼1.0 nm) from the membrane, and we removed the extra water molecules from the system in the last configurations, leaving a distance of ∼1.6 nm to the periodic images in the *Z* axis. This protocol leads to the removal of half the number of water molecules to ∼20k. Since the CpHMD simulations are more computationally demanding, this system reduction step was essential to increase their computational performance. The final system configurations were then subject to normal minimization and initialization protocols (see below).

### 2.2 MM/MD Settings

All simulations were performed using GROMACS 5.1.5 package^27^ and the GROMOS 54A7 force field.^28^ All systems were energy-minimized in a two-step procedure with 10k integrator steps each, using the steepest descent algorithm for both, the first one being unconstrained and the second one with p-LINCS and SETTLE constraining algorithms applied on all bonds to the solute and solvent, respectively. The initialization protocol consisted of 200 ps of NVT MD with the v-rescale thermostat^29^ set for a reference temperature of 310 K, with a temperature coupling of 0.01 ps. Starting velocities were assigned from a Maxwell distribution representative of 310 K. This was followed by 200 ps of NPT MD with the Parrinello–Rahman isotropic barostat (semi-isotropic for the membrane systems)^30^ set for a reference pressure of 1 bar and pressure coupling of 1 ps. Both of these steps used an integration time of 1 fs. The long-range electrostatics were treated with the reaction field method,^31^ with a group cutoff scheme of 1.4 nm, and the neighbor list was updated every 10 steps. van der Waals interactions were simply truncated above 1.4 nm. The integration step was updated to 2 fs in all subsequent MD steps.

The protocol that uses PyMOL to generate the initial dendrimer conformations inevitably introduces an initial bias that needs to be removed before proceeding. We followed a previously developed method^13^ that, after the NPT initialization step, used an MD simulation (1 ns) with the charge of every dendrimer atom changed to +0.05. This approach leads to a small charge repulsion between all atoms that promotes the stretching of the structure and eliminates any specific bias (Figure S1 of the Supporting Information). After this step, the structure was relaxed with a final MD segment (200 ps) with regular charges. For production, we updated the v-rescale temperature coupling to 0.1 ps and the barostat pressure coupling to 2 and 5 ps for water and membrane simulations, respectively.

### 2.3 Poisson–Boltzmann/Monte Carlo and CpHMD Settings

We used our implementation of the CpHMD method^32−35^ that couples the conformational sampling of MD simulations with the protonation state sampling provided by Poisson–Boltzmann/Monte Carlo (PB/MC) calculations. In our method, a CpHMD cycle is iterated and starts with a PB/MC step, where we calculate the free energies of each protonation state for our titrating groups, using the program DelPhi V5.1^36^ with G54A7 atom partial charges and the radii calculated from the same force field Lennard-Jones parameters at 2 RT.^37^ Unlike rigid-body p*K*_a_ calculations, where the dielectric constant is increased to mitigate the lack of conformational flexibility, in CpHMD, all calculations are performed with a low dielectric constant in the solute (dendrimer and membrane) and rely on the MM/MD to sample the correct conformational ensemble. We use a dielectric constant of 2 (instead of 1) to address the lack of polarization in the PB model. The water solvent is treated implicitly and with a high dielectric constant (80), since it has been shown that the inclusion of explicit water at the solute interfaces does not have a significant impact on the ability to predict p*K*_a_ values.^37^ The molecular surface of the solute was defined by using a probe with a radius of 1.4 Å. The populations of each protonation state are sampled using PETIT, which uses an MC scheme on the free energy terms calculated from the previous PB step.^38^ A total of 10^5^ MC cycles were performed for each conformation, with the protonations of the last cycle selected as the new protonation states. The next step in the CpHMD cycle is a short solvent relaxation MD step of 0.2 ps, with a frozen solute, to allow the solvent molecules to adjust to the new protonation states. The cycle is then completed with a segment of 20 ps of production MD, to sample the conformational space of the system with the new protonation states in the solute.

For the water simulations, we simulated a total of 5 replicates per pH value and 10 pH values (150/200 ns each), ranging from 3 to 12 with a 1 pH unit step. At low pH values, we extended our simulations to 200 ns to ensure that the dendrimer structural properties were properly equilibrated. Nine systems, namely MH18 and MH13, their respective full d-amino acid variants, DMH18 and DMH13, MH18D1, MH18D2, MH18D3, and MH18D4, four variants of the MH18 sequence with different combinations of d- and l-amino acids, and MH47, identical to MH18 except for having the titrating lysines from G2 mutated to leucines (Table 1).^7^

**Table 1:** List of Dendrimer Sequences Simulated with the Experimental Data on RNA Binding, Cellular Uptake, and siRNA Silencing Activity^a^

general information		experimental data		
system	sequence	free siRNA (%)	cellular uptake [GMFI]	GAPDH activity [%]
MH18	(KL)_8_(*K*KL)_4_(*K*LL)_2_*K*LLLL	4.8 ± 0.4	67 ± 9	28 ± 9
DMH18	(kl)_8_(*k*kl)_4_(*k*ll)_2_*k*llll	4.6 ± 0.7	94 ± 6	28 ± 9
MH18D1	(kl)_8_(*K*KL)_4_(*K*LL)_2_*K*LLLL	9.1 ± 1.1	26 ± 2	54 ± 6
MH18D2	(KL)_8_(*K*KL)_4_(*K*LL)_2_*K*llll	4.6 ± 0.3	61 ± 2	70 ± 20
MH18D3	(KL)_8_(*k*KL)_4_(*k*LL)_2_*k*LLLL	18.8 ± 3	37 ± 3	98 ± 8
MH18D4^b^	(kl)_8_(*K*KL)_4_(*k*ll)_2_*K*LLLL			
MH47	(KL)_8_(*K*LL)_4_(*K*LL)_2_*K*LLLL	3.0 ± 0.1	238 ± 8	77 ± 14
MH13	(KL)_8_(*K*KL)_4_(*K*LL)_2_*K*K(C_16_)K(C_16_)	4.8 ± 0.7	98 ± 14	32 ± 5
DMH13	(kl)_8_(*k*kl)_4_(*k*ll)_2_*k*k(C_16_)k(C_16_)	3.9 ± 0.5	174 ± 30	31 ± 2

a One-letter code for amino acids was used, where uppercase letters represent l-amino acids, and lowercase letters represent d-amino acids. Italic letters represent branched lysines. All experimental data was obtained from ref (7). Free siRNA is inversely proportional to dendrimer binding to RNA, cellular uptake is a measure of endocytosis at pH 7.4, and GAPDH is the enzyme activity that should correlate inversely to endosome evasion.

b This dendrimer sequence was only built computationally, hence, it has no corresponding experimental data.

This protocol allowed us to perform a full pH titration of all of the dendrimers. Since only one copy of the dendrimer is present in the simulation box, our model assumes the typical infinite dilution setup, which is typical of concentrations under self-aggregation conditions. In the membrane simulations, we performed a total of 10 replicates per pH value (150 ns each) and 4 pH values, 4.5 to 7.5 with a step of 1 pH unit. We focused our study on only four systems, namely MH18, MH13, MH18D3, and MH47.^7^ Since the membrane simulations are very computationally demanding, we focused on the most promising dendrimer sequences and on a pH range that is physiologically relevant, like the cell exterior (pH 7.5), the late endosome (pH 5.5), and lysosomes (pH 4.5).

### 2.4 Analyses

All analyses were performed by using the GROMACS 5.1.5 software package and in-house tools. All structures were visualized and rendered using PyMOL, and all plots were plotted using gnuplot. The average protonation states of each titrating group were fitted to the Hill equation, which allowed us to estimate their p*K*_a_ values. All error values presented were calculated using the standard error of the mean (SEM), except for the errors regarding the estimated p*K*_a_ values. For these, we estimate the errors using the Jackknife method,^25^ which consists of a leave-one-out approach to resample our data and provide more robust data sets to the Hill fitting procedure.

#### 2.4.1 Root Mean Square Deviation

Since the root mean square deviation (rmsd) calculation requires the performance of a structural fit, the symmetry (or pseudosymmetry) of a given system may have an impact on the calculated values, causing an artificial increase in the resulting rmsd value. In these systems, this problem is quite evident, since each branch of the dendrimer can effectively rotate and switch places with a pseudosymmetric branch, which can influence the calculated rmsd values. Hence, the rmsd for each permutation of the dendrimers, which amounts to 2^7^ = 128 different combinations, was calculated for each conformation and the lowest value was selected as the real rmsd. Additionally, the typical rmsd calculations using the initial structure as a reference are meaningless as the starting conformations are not representative of the final ensembles. To circumvent this, we calculated the rmsd values using a central structure as ref (13). This is calculated from the cross rmsd values among structures from all systems except for MH47, all pH values, and replicates and by selecting the one with the lowest sum of cross rmsd values. Since this step is very computationally expensive, we only used the final structure of all simulations, leading to a total of 400 eligible structures.

#### 2.4.2 Energy Landscapes

The conditional free energy landscapes were calculated from a probability density function of a two-dimensional space using rmsd and Radius of Gyration (*R*_g_) as structural coordinates. These are two of the best properties that capture the size of dendrimers and the conformations that fold in distinct ways. The probability density functions were estimated using a Gaussian kernel estimator, with grids of 0.009 Å^3^.^13^ The conditional energy (*E*_(r)_) surfaces were computed with the following equation
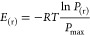
1where *R* and *T* are the ideal gas constant and temperature, respectively, while *P*_(r)_ and *P*_max_ are the probability density function, and its respective maximum.

#### 2.4.3 Sphericity and Shape

Another useful property to characterize the conformational space of dendrimers is their sphericity, which is derived from the asphericity and provides a measure of the deviation from a perfect sphere and can be calculated using the moments of inertia, with the following equation
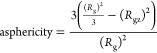
2where *R*_g_ is the radius of gyration of a given molecule, and *R*_gz_ is the shortest moment of inertia.

Since this property fluctuates between 1 and 0, where 1 corresponds to a structure completely deviated from a sphere and 0 corresponds to a perfect sphere, we can invert this property and obtain the sphericity percentage by applying the following transformation
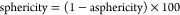
3

This property only quantifies the deviations from a perfect sphere without providing information about the overall shape of the dendrimer. Another property, also derived from the moments of inertia, is the approximation to an oblate- or prolate-like shape. To quantify this property, we need to calculate the average between the shortest and longest moments of inertia and compare it with the intermediate moment of inertia. If this intermediate value is closer to the longest or the shortest moment of inertia, we obtain that the molecule is more akin to an oblate-like shape (like a Frisbee) or a prolate-like shape (like a rugby ball), respectively. We calculate this with the following expression
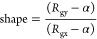
4where *R*_gy_ and *R*_gx_ correspond to the intermediate and the longest moments of inertia respectively, while α corresponds to the average between the longest and the shortest moments of inertia. In summary, our dendrimer structures will show positive shape values for oblate-like conformations and negative values for prolate ones.

#### 2.4.4 Deformation

The local deformation of the membrane (in the vicinity of the dendrimers) and the radial thickness profiles were computed using MembIT.^39^ For both cases, lipids more than 15 Å away from the dendrimer are considered to be unperturbed (bulk) and are used as a reference. For the local deformation calculation, only lipids within 6 Å of the dendrimer were considered. For the monolayer thickness profiles, radial slices of 1 Å, with an increment/step of 0.5 Å, were defined to a maximum distance of 25 Å.

#### 2.4.5 Orientation

The orientation of the dendrimer can provide hints about whether the hydrophobic core is turned toward the water phase or the membrane. To obtain this orientation, we calculate the geometry centers of the dendrimer hydrophobic cores (G0) and of the remainder of the dendrimers (G1 + G2 + G3). Then, the z component (in absolute value) of the G0 geometric center is subtracted from the Z component (in absolute value) of the rest of the dendrimer. Hence, if this property is positive, then the hydrophobic core is turned toward the membrane; otherwise, it is turned toward the aqueous phase.

#### 2.4.6 Protonation Analysis

The protonation of each titrating group was analyzed individually, and the titration curves for the simulations in water were also calculated for each type of titrating group in each generation of the dendrimers, taking advantage of the pseudo symmetry of these systems. In the membrane simulations, due to a lack of sampling for the interaction process between the dendrimer and the membrane, we computed conditional p*K*_a_ profiles, by calculating the p*K*_a_ values along the membrane insertion vector.^25,33^ Similarly, we also calculated protonation profiles to estimate how the different groups change their total charge when inserted into the membrane.

## 3 Results and Discussion

### 3.1 Peptide Dendrimer Simulations in the Water Phase

We performed CpHMD simulations of several peptide dendrimer systems in water and started by evaluating their equilibration. For this, we followed the time series of the system total charge, the radius of gyration (*R*_g_), the rmsd, and the dendrimer sphericity (Figures S2–S5 of Supporting Information). Although protonation and conformation are properties strongly coupled, influencing each other, the system total charge usually equilibrates relatively fast (Figure S2 of the Supporting Information). The rmsd and the sphericity also seem to converge within the initial 50 ns. Given the typical lack of secondary structure motifs in peptide dendrimers, the *R*_g_ property is particularly useful since it provides a good measure of the molecule’s size and overall structure.^17^ The *R*_g_ time series confirm that this is the most difficult property to equilibrate (Figure S3 of Supporting Information). At high pH values, we observe convergence within the first 50 ns; however, at lower pH values, at least 100 ns were needed. Therefore, extended CpHMD simulations (200 ns) were required to ensure the same sampling (100 ns) at all pH values.

**Figure 2:**
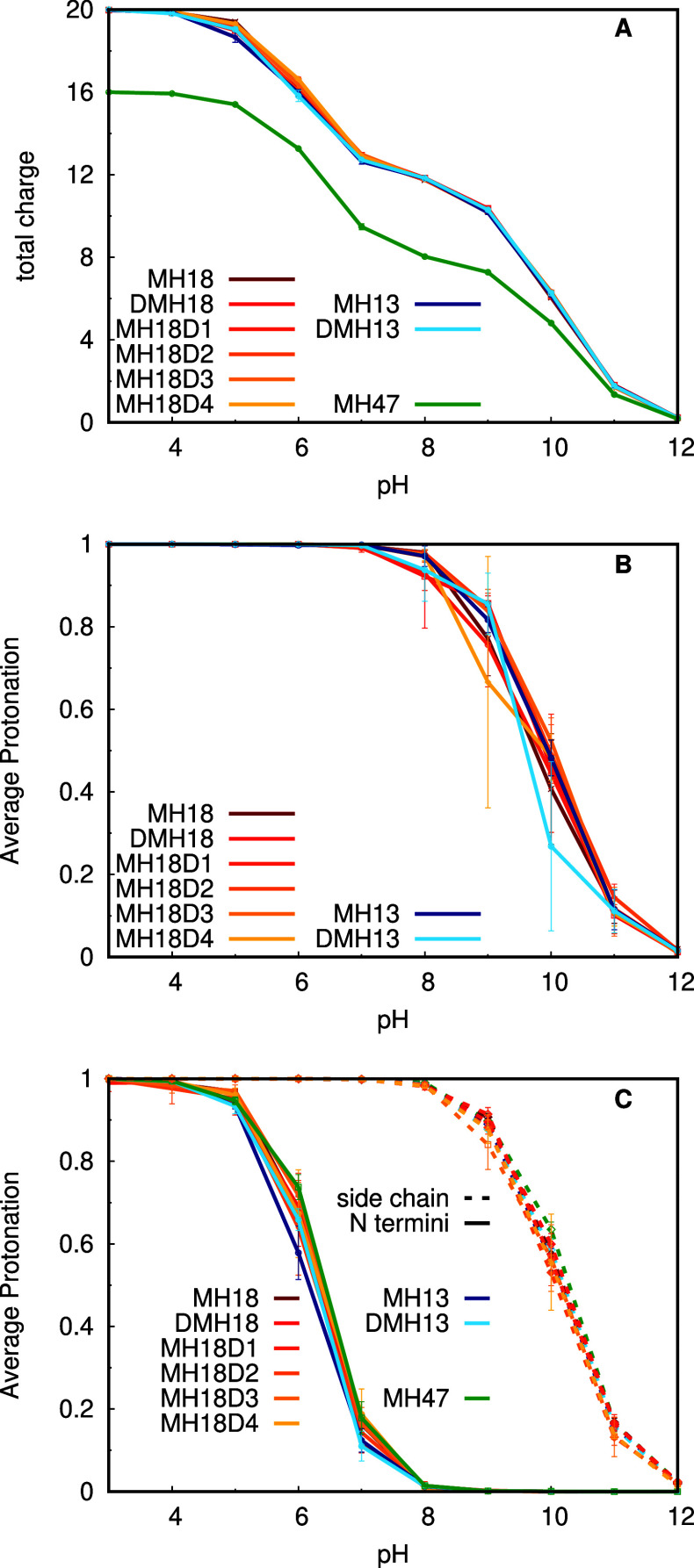
Total titration curves (A) and titrations per generation, G2 (B) and G3 (C). In G2 only Lys residues are present, while in G3, the titration curves were split into Lys residues and N-termini. All systems are represented, with MH47 in green, MH13 systems in a blue gradient, and MH18 systems in a red-yellow gradient.

After computing the dendrimers’ titration curves (Figure 2A), we observe that all systems display identical and indistinguishable behavior, except for MH47, obviously due to the absence of those 4 lysine residues that were mutated to leucines. This result indicates that the different chiral changes and the nature of the hydrophobic core have no meaningful impact on the pH dependence of these dendrimers. The total titration curves show two clear transitions around pH 6.5 and 10, which correspond to the titration of the N-termini and amino groups of the Lys side chains, respectively. It is also interesting to note that these dendrimers change significantly their protonation states at even the most acidic pH values studied (Table S1 of Supporting Information), which highlights the importance of using CpHMD methodologies, in contrast with conventional MD, to study these systems. When decomposing the titration curves per dendrimer generation, we also noticed that the Lys residues in G2 and G3 behave quite similarly (Figure 2B,C), indicating that lysine residues in G2 are not significantly buried in these dendrimers, as could be expected.

**Figure 3:**
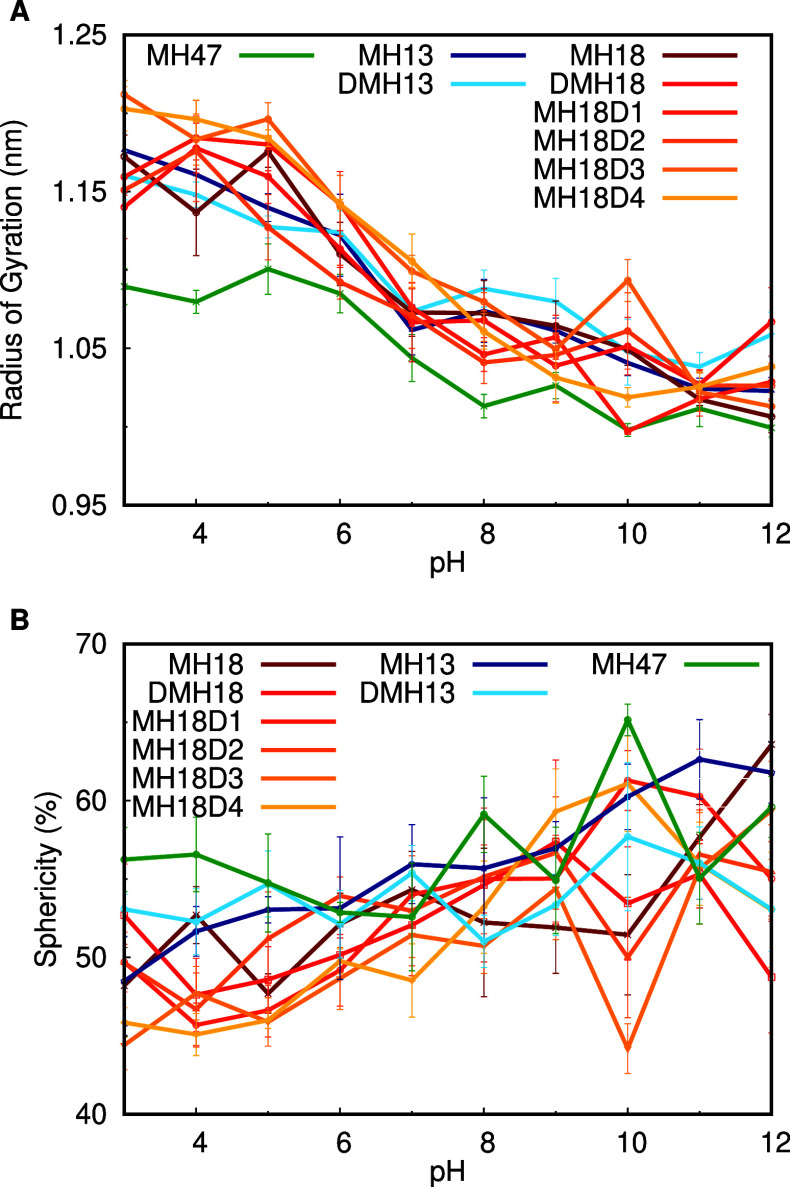
Radius of gyration (A) and sphericity (B) over pH. All systems are represented, with MH47 in green, MH13 systems in a blue gradient, and MH18 systems in a red-yellow gradient.

Regarding the dendrimers’ structure, we observed a decrease in their overall size (*R*_g_), accompanied by an increase in sphericity at higher pH values (Figure 3). This is easily explained by the fact that increased pH leads to deprotonation of the charged residues, namely, the N-termini and the lysines, promoting less charge repulsion. As a result, the dendrimers tend to collapse, acquire a more compact shape (smaller volume), and become more spherical. Another noteworthy piece of evidence is that most systems display a similar pH dependence for both the radius of gyration and the sphericity. The main exception to this is MH47, which has 4 fewer Lys residues and, hence, exhibits a smaller charge repulsion at lower pH values. The dendrimer sphericity provides a measure of similarity to a sphere, and since all dendrimer systems only reach ∼65% at very high pH values, it suggests that these molecules are not as globular as most proteins. We also observed that the dendrimers’ overall shape is very dynamic, which is supported by the large fluctuations observed in their sphericity time series (Figure S5 of Supporting Information).

**Figure 4:**
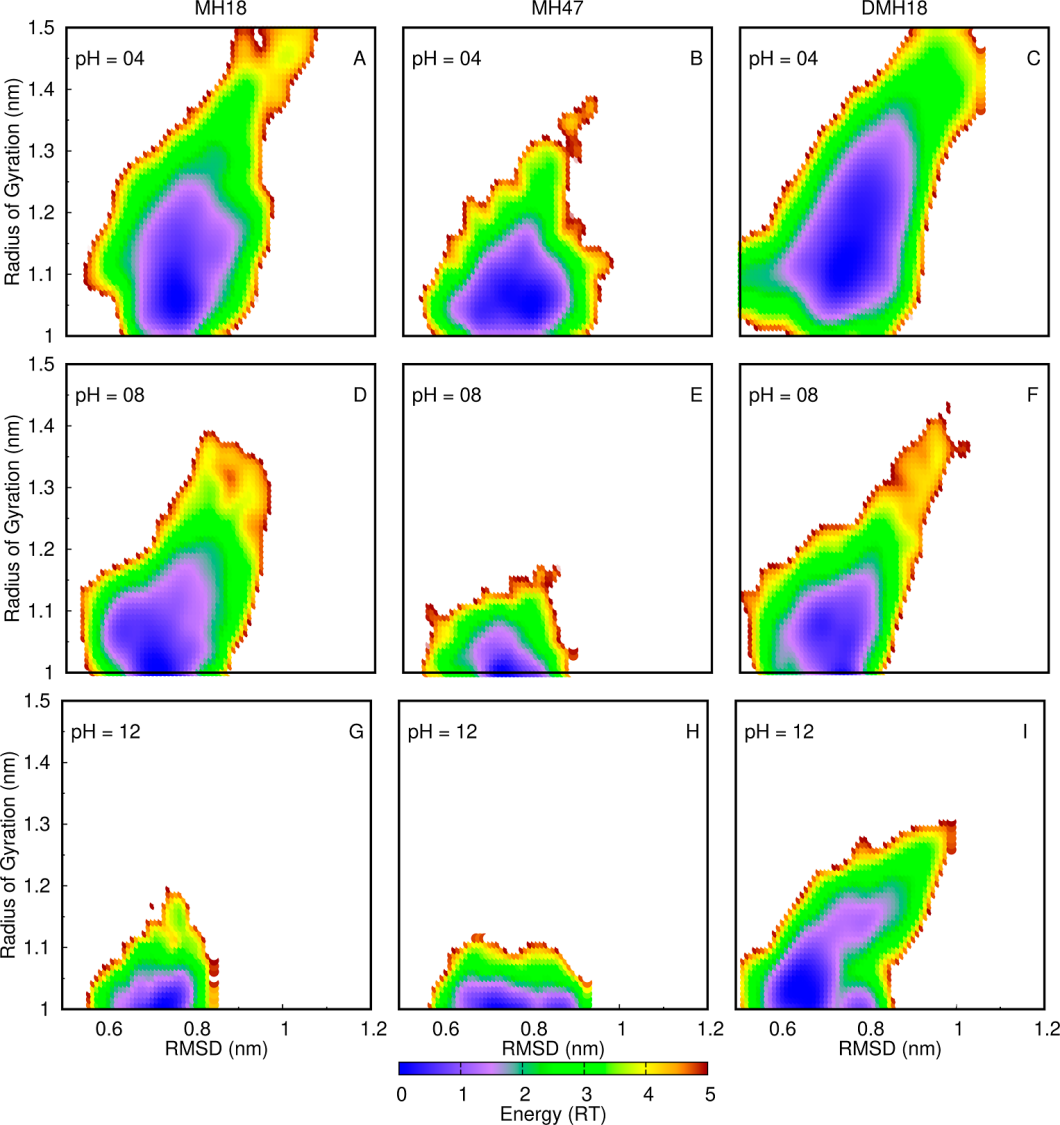
Energy landscapes of the radius of gyration vs the rmsd for MH18 (A,D,G), MH47 (B,E,H), and DMH18 (C,F,I) at pH 4.0 (A–C), 8.0 (D–F), and 12 (G–I). The energy scale is shown as a color palette at the bottom.

In an attempt to identify structural features within these dendrimers that are influenced by pH, we calculated energy landscapes from the probability density of a 2D surface using both the radius of gyration and the rmsd as structural coordinates (Figure 4). These energy landscapes show a funnel-like shape with little structure. The overall size of the minima narrows with the increase of pH, as a result of the decrease in the radius of gyration both in absolute values and in their spread. We do observe a gain in structure, more pronounced at higher pH values, with the formation of two small clusters. This is very clear for DMH18 at pH 12, where two clear minima are distinguished: one at rmsd 0.6–0.7 nm and another one at 0.8–0.9 nm. We can assign these clusters to two different topological arrangements of the dendrimer branches, one where four branches are detached from the remaining four (lower rmsd values) and another one where two branches are detached from the remaining six (higher rmsd values) (Figure S6 of Supporting Information). The difference in rmsd values results from the fact that the reference (central) structure also has a 4:4 topology arrangement.

Overall, we observe a high homogeneity between the different dendrimer topologies in the water phase, in terms of both protonation and their structure. The main exception is for MH47, which is different from the remaining sequences, bearing four fewer Lys residues. Notwithstanding, these similarities do not provide a clear explanation for the distinct experimental performances of the different dendrimers, including the different l/d amino acid compositions. It is expected that the differences in chirality within our peptide dendrimers could be potentiated when these molecules interact with other chiral molecules.

### 3.2 Peptide Dendrimer Interactions with a Membrane

**Figure 5:**
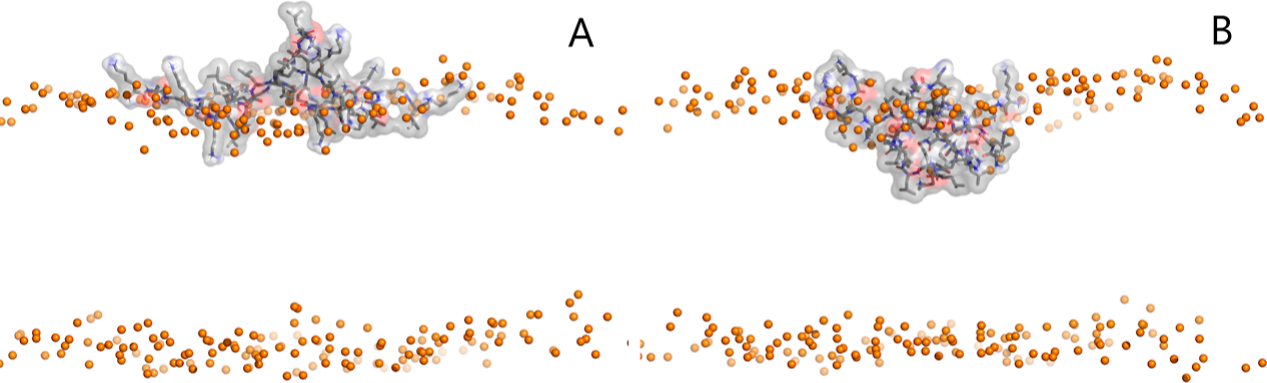
Representative structures of the MH18 dendrimer at pH 7.5 (A) and 4.5 (B). The membrane is represented only by the phosphorus atoms (orange spheres) for clarity. The dendrimer is depicted with sticks and a transparent surface, colored gray.

We also performed CpHMD simulations of a subset of the previously simulated dendrimers, namely, MH18, MH18D3, MH13, and MH47, interacting with a membrane bilayer. To assess the simulations’ equilibration, we evaluated the membrane insertion of these dendrimers (Figure S7 of Supporting Information), which is a difficult property to equilibrate within our time scale. In most simulations, the dendrimers seem to adsorb to the membrane in the initial 50 ns, with only one detach and readsorption event happening at pH 7.5 for MH18D3. From these time series, we also note a trend where the dendrimers adsorb/insert deeper into the membrane at lower pH values (Figure 5). The protonation and many structural properties are greatly affected by the membrane insertion level. Hence, we calculated them along the membrane normal and presented the results as a function of the membrane insertion. There are many structural properties of the dendrimers, such as the radius of gyration, sphericity, oblate/prolate shape, and orientation along the membrane normal, as well as the membrane area and its local deformation that seem to equilibrate relatively fast (Figures S8–S13 of Supporting Information), once the dendrimer adsorbs to the membrane (∼50 ns).

**Figure 6:**
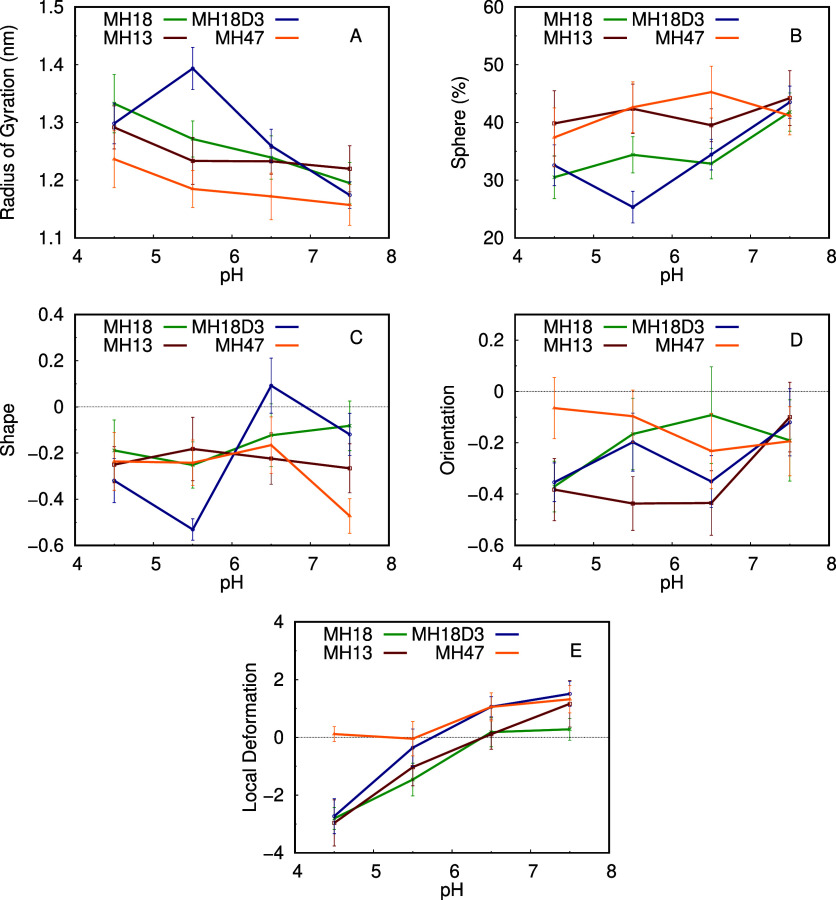
Dendrimers radius of gyration, sphericity, oblate/prolate shape, average orientation along the membrane normal, and local membrane deformation over pH. MH18 is shown in green, MH18D3 in yellow, MH18D3 in yellow, and MH47 in red.

Analyzing these properties over pH, we notice that the dendrimers’ radius of gyration, sphericity, shape, and orientation are not substantially affected (Figure 6A–D). Nevertheless, we cannot exclude the possibility that pH induces small structural differences that we have not captured on the short time scale of our simulations. The sampling limitations are common in membrane simulations since we need to sample the peptide dendrimer/membrane configurational space on top of the conformational space of the dendrimers, which is itself a challenge. We mitigated these limitations by performing 10 × 150 ns simulations at each pH value, which resulted in a reasonable level of convergence and, consequently, error bars that allowed us to identify trends and, sometimes, some clear differences in the properties. There is a trend of a decrease in the radius of gyration and an increase in sphericity with the pH increase. This can be interpreted by the charge decrease of the dendrimers that promotes their compactness and globular shape. There is also an effect induced by the presence of the membrane compared to the behavior in water. When adsorbing to the membrane, the dendrimers tend to stretch on its surface and not to compact as much, which leads to a higher radius of gyration and a smaller sphericity (Figure S14 of Supporting Information). We also see that the dendrimers are preferably shaped as a prolate with the hydrophobic core facing away from the membrane, but there are hardly any trends in their pH profiles. On the other hand, there is a clear effect of pH on the extent of membrane deformation induced by the dendrimers (Figure 6E). At low pH, we observe a pronounced membrane deformation with all dendrimers, excluding MH47, most likely due to its lower overall charge.

**Figure 7:**
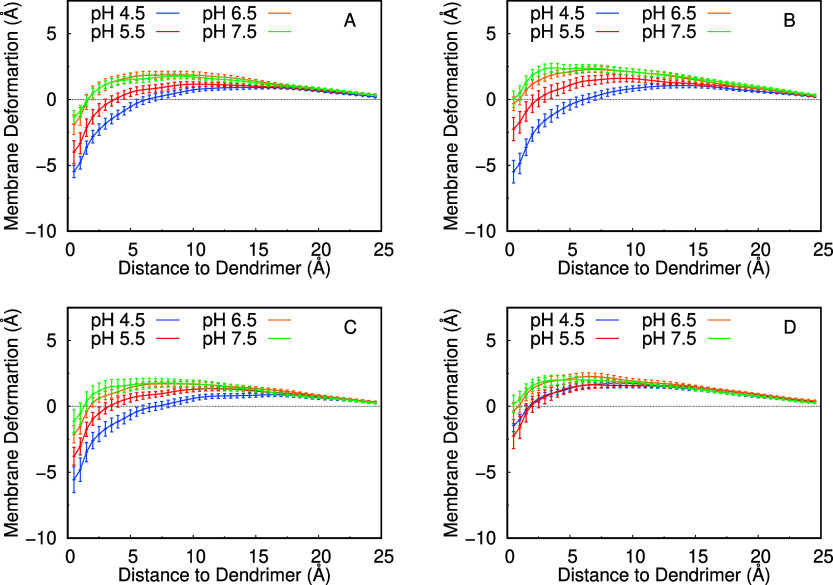
Membrane deformation profiles of MH18 (A), MH18D3 (B), MH13 (C), and MH47 (D) at different pH values. The lipids beyond 25 Å were considered unperturbed (bulk). For more details on the calculation, see the Methods section.

The membrane deformation induced by the dendrimers seems to be the most sensitive property observed. Therefore, we calculated complete membrane deformation profiles for all dendrimers and pH values (Figure 7). As hinted by the local deformation (Figure 6E), we observe a strong pH-dependent perturbation in the lipids that interact more directly with the dendrimers. Higher (positively) charged dendrimers seem to insert more deeply into the membrane, dragging inward (∼6 Å) many of those interacting lipids toward the membrane center (Figure 5B). These effects are quite dominant since they are promoted by strong electrostatic interactions between the dendrimers’ ammonium groups and the lipid phosphate groups. The lipids located laterally on the second and third coordination spheres of the dendrimer are also perturbed, slightly protruding from the bilayer. This effect was also induced by electrostatic interactions of those lipids with dendrimer ammonium groups that are located far from the membrane. Since these dendrimers are constituted by hydrophobic and polar (often charged) residues, there is an amphiphilic nature on their surface that favors interactions with phospholipids and perturbs the overall stability of the lipid bilayer.

**Figure 8:**
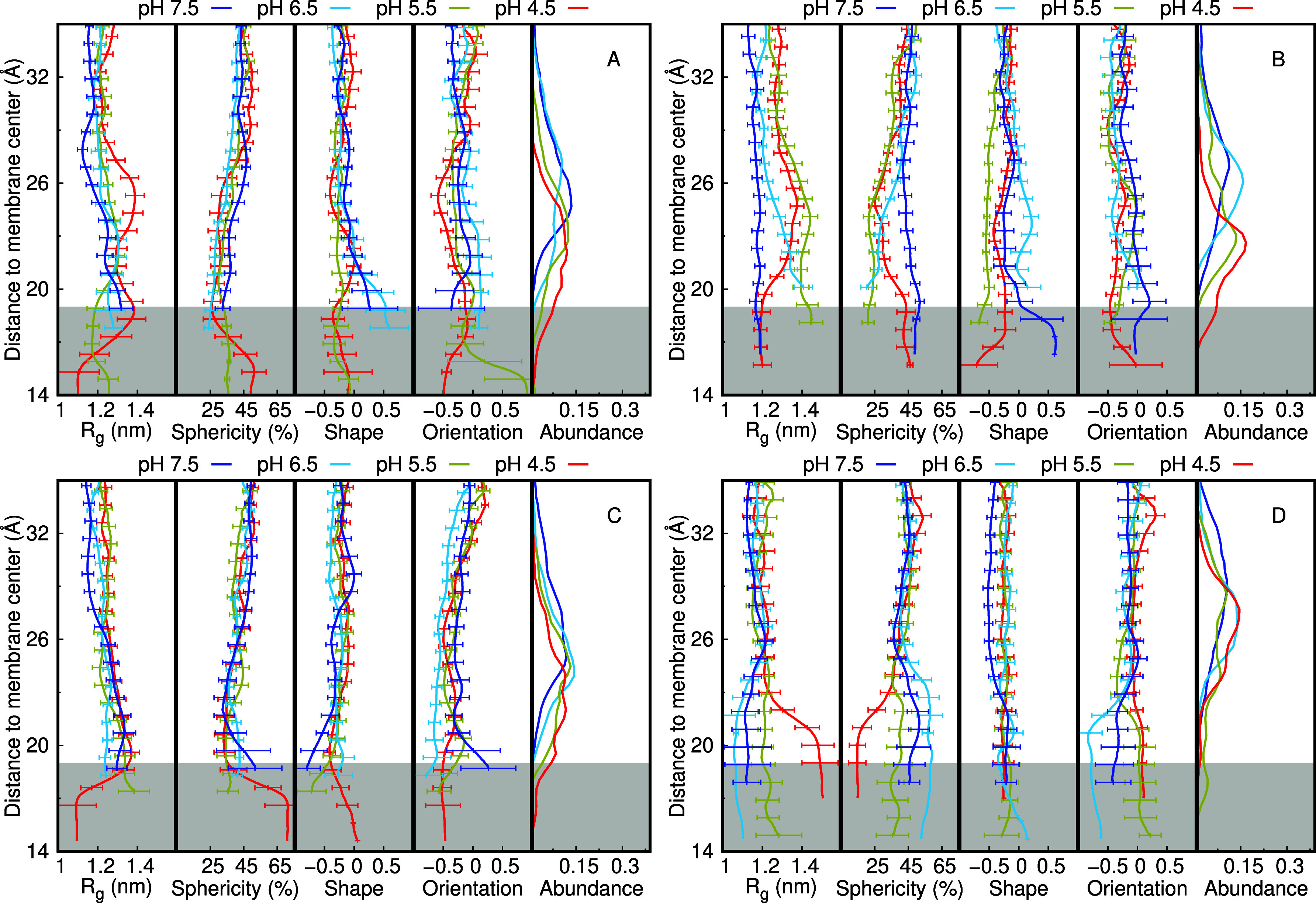
Dendrimers structural properties over distance to membrane center for MH18 (A), MH18D3 (B), MH13 (C), and MH47 (D). The pH values 4.5, 5.5, 6.5, and 7.5 are shown in red, olive, cyan, and blue, respectively. The histogram distributions of the distances to the membrane center at each pH value are also shown on the right side of the plots. The gray-shaded region corresponds to the membrane interior, assuming an unperturbed membrane (monolayer thickness of ∼19 Å). The distance to the membrane center in these plots was calculated using the geometric center of the dendrimer and not the membrane insertion of a specific group. The error bars were calculated using the SEM at each slice of the distance to the membrane center. Slices with inadequate sampling (<400 points) were discarded.

The peptide dendrimer structural properties that we studied over pH (*R*_g_, sphericity, shape, and orientation) can still be deconvoluted along the membrane normal (Figure 8). For most cases, as previously discussed, there are no significant differences in the four pH values studied, with small divergences appearing only in regions with a dwindling sampling. The main impact of pH (and protonation) is captured in the insertion abundance histograms (right subplots of Figure 8), where more protonated dendrimers tend to access deeper regions of the membrane, probably due to the membrane deformation observed previously.

**Figure 9:**
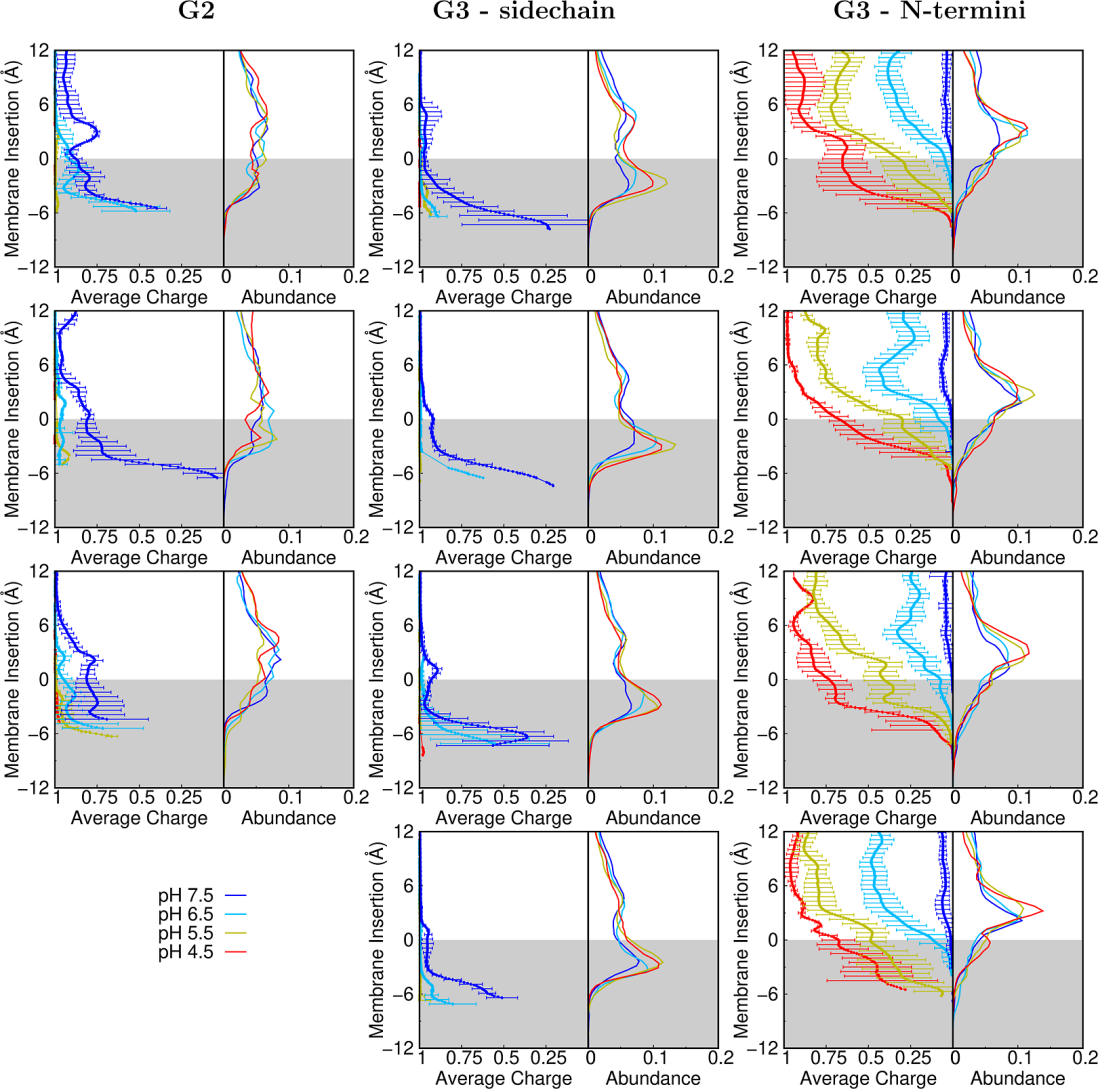
Dendrimer protonation profiles over membrane insertion for MH18 (first row), MH18D3 (second row), MH13 (third row) and MH47 (fourth row). All different titrating group types are shown, namely, side chains of lysines from G2 (left column), side chains of lysines from G3 (middle column), and N-termini of lysines from G3 (right column). pH values 4.5, 5.5, 6.5, and 7.5 are shown in red, olive, cyan, and blue, respectively. The error bars were calculated using the SEM at each slice of insertion. Slices with less than 1500 points were discarded due to the lack of sampling.

**Figure 10:**
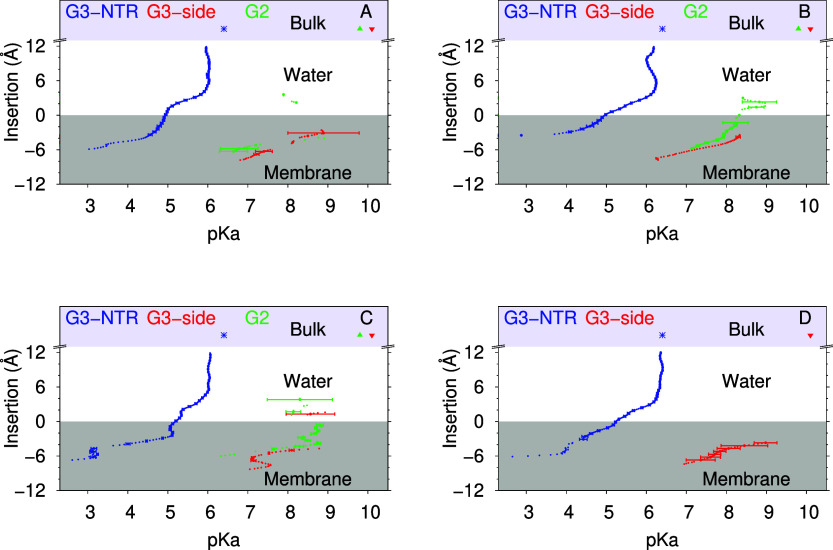
Dendrimer p*K*_a_ profiles over membrane insertion for MH18 (A), MH18D3 (B), MH13 (C), and MH47 (D). The G2 Lys, G3 Lys, and N-termini are shown in green, red, and blue, respectively. The water p*K*_a_ values for each of these groups are represented as points with matching colors in the Bulk region, on the top of the plots and shaded as light blue. The error bars were calculated using the jackknife method at each slice of insertion. Slices with inadequate sampling (<2500 points) were discarded.

The protonation states of the peptide dendrimers are influenced by pH. Notwithstanding, the strong effects due to the lipid interactions may perturb significantly the behavior previously observed in water. Given the pH range simulated, from 4.5 to 7.5, and considering the experimental p*K*_a_ value for the Lys side chain in solution (10.4^40^), we would expect that all Lys residues in our dendrimers (G2 and G3) to only titrate when inserted in the membrane and at higher pH. The membrane insertion leads to water desolvation which stabilizes the deprotonated forms, lowering significantly the p*K*_a_ values of cationic residues,^33^ resulting in, at least, some deprotonation for these groups (Figure 9). Another consequence is that the p*K*_a_ calculations of our Lys residues are often extrapolations and estimated by using data from only the two higher pH values (Figure 10).

On the other hand, the titration of the N-termini is well captured as well as its behavior when modulated by the membrane, leading to a more complete p*K*_a_ profile for this group along the lipid bilayer insertion. Identically to what happens to the Lys side chains, desolvation also stabilizes its deprotonated form, resulting in profiles that start from at least partially protonated dendrimers in the water phase and, due to water desolvation, end up completely deprotonated at deeper insertions, even at very low pH values. Some profiles show small deviations at the water/membrane interface. For example, MH18 at low pH shows a decrease in the average protonation between 6 and 2 Å of insertion (Figure 9), which can be explained by charge repulsion from a direct interaction with the choline groups of the lipids. Around 2 and −2 Å, the tendency is to become more protonated, most likely due to the interaction with the negatively charged phosphate groups. Similar trends can be observed for other dendrimers at certain pH values; however, in most cases the fine detail of the interactions with the different lipid regions is lost or convoluted in the averaging procedure performed in this slicing protocol. The final p*K*_a_ profiles calculated for the N-termini of all residues (Figure 10) follow the standard profile already reported for the Ala pentapeptide,^33^ which suggests that these groups are not involved in intramolecular interaction significantly different from a simple peptide.

## 4 Conclusions

In this study, we observed that the conformational space of the different peptide dendrimers in water is mainly affected by the solution pH, where the acidity leads to an increase in the molecules’ size. Since this dependence is proportional to the number of titrable residues, the dendrimer with fewer Lys residues (MH47) seems to expand less at lower pH. The different compositions of l/d amino acid and the substitution of the tetra-leucine core (MH18) for two lipidic tails (MH13) did not influence significantly the conformations nor the titration curves of the dendrimers. The lack of an effect on the stereoisomers was somewhat expected since the changed chiral centers in our peptide dendrimers will induce a different behavior only when these molecules interact with other chiral molecules, like lipids and/or nucleic acids.

In the membrane systems, we aimed to investigate how the different dendrimers interact with the lipid bilayer and what role acidity plays in destabilizing the membrane, mimicking the endosome evasion process. We selected 4 different dendrimers (MH18, MH18D3, MH13, and MH47), focused on the acidic to neutral pH range (4.5–7.5), and circumvented most sampling limitations by studying the protonation and conformation of these molecules along the membrane interaction/insertion pathway. Our results helped to characterize in detail the membrane-induced deprotonation phenomena in all dendrimers, which was significant in the N-termini groups but only partial in the Lys side chains. This process seems to be independent of the dendrimers’ topology and total charge, leading, for example, to p*K*_a_ profiles that are very similar to those of Ala pentapeptides^33^ or Lewis-base-containing drugs.^41^ Although all dendrimers followed a similar membrane adsorption process, especially around neutral pH, at lower pH, we observed membrane insertion and destabilization in some cases. Indeed, MH18, MH18D3, and MH13, at pH 4.5 and partially at pH 5.5, showed significant membrane insertion and deformation, similar to what has been observed in MD simulations of many cationic peptides.^42−45^ On the other hand, MH47, which has 4 fewer positive charges, showed no ability to perturb the membrane, even at pH 4.5. This limitation in triggering the initial steps of membrane disruption may be the reason behind MH47’s poor performance in the siRNA endosome evasion process.^7^

Overall, we showed that despite the similarities in the conformational space of all peptide dendrimers studied in water, the amount of charge in specific pH ranges and the specific details of how those charges interact with a lipid bilayer can lead to important distinctions in their mode of action. The ability of these dendrimers to destabilize the membrane upon acidification is a hallmark of the DNA/RNA transfection mechanism. In addition, the molecular details provided by this work will benefit future research in developing new scaffolds and topologies for more effective dendrimers. Our future work will focus on studying the role of these dendrimers in RNA binding and stabilization, the pH dependence of this process, and the impact of the resulting conjugates on the lipid bilayer.

## Data Availability

The GROMACS package is freely available software used to perform MD simulations and can be downloaded at https://manual.gromacs.org/documentation/2020.1/download.html. PyMOL v2.5 is also free software for molecular visualization and generating high-quality images. It can be downloaded from https://pymol.org/2. A zip file with all topologies, system configurations, parameters files, and the CpHMD code is also provided.

## Supporting Information Available

Time series with total charge, radius of gyration, rmsd, and sphericity in water for all dendrimer systems; representative conformations of the DMH18 dendrimer, at pH 12 highlighting the low and high rmsd basins; time series of the distance to the center of the membrane, membrane area, radius of gyration, local membrane deformation, sphericity percentage, membrane orientation, and shape for dendrimers MH18, MH18D3, MH13, and MH47; radius of gyration and average sphericity for MH18 and MH47 in water and membrane simulations (PDF)
