# Supplementary material for: High Charge Density in Peptide Dendrimers is Required to Destabilize Membranes: Insights into Endosome Evasion
Source: J Chem Inf Model. 2024 Apr 8;64(8):3430–42. doi: 10.1021/acs.jcim.4c00018 (PMC11040734; doi:10.1021/acs.jcim.4c00018)
Supplement: Supplementary file 1 — ci4c00018_si_001.pdf [file ci4c00018_si_001.pdf]

Supporting Information: High Charge Density in Peptide Dendrimers is Required to Destabilize Membranes: Insights into Endosome Evasion

Filipe E. P. Rodrigues,[†] Tamis Darbre,[‡] and Miguel Machuqueiro[*,†]

†*BioISI – Instituto de Biossistemas e Ciências Integrativas, Faculdade de Ciências, Universidade de Lisboa, 1749-016, Lisboa, Portugal*

‡*Department of Chemistry, Biochemistry and Pharmaceutical Sciences, University of Bern, Bern, Switzerland*

E-mail: machuque@ciencias.ulisboa.pt

Phone: +351-21-7500112

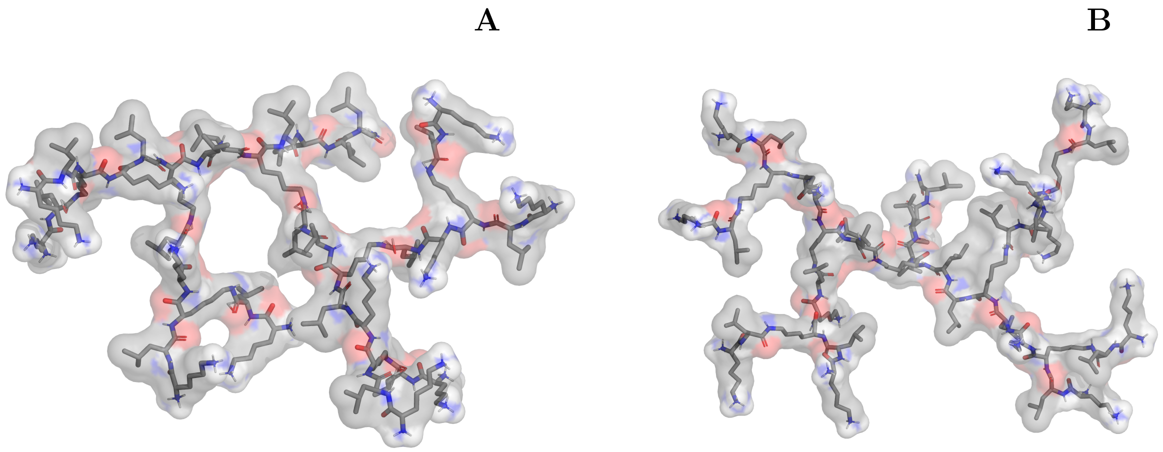

Figure S1: Molecular representations of MH18 dendrimer after construction in PyMOL (A) and after the conformation uniformization step of our initialization protocol (B).

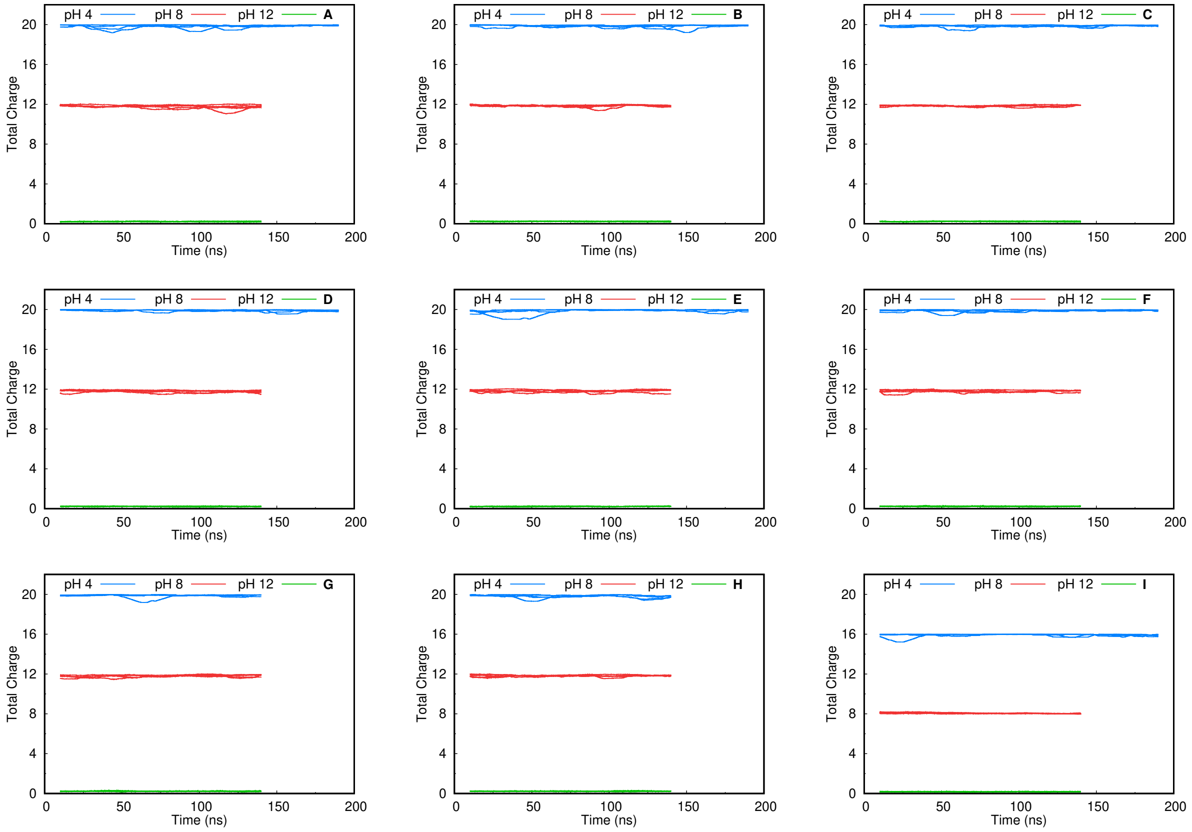

Figure S2: Total charge of all systems, namely MH18 (A), DMH18 (B), MH18D1 (C), MH18D2 (D), MH18D3 (E), MH18D4 (F), MH13 (G), DMH13 (H) and MH47 (I) in water and over time. Five replicates of acidic (pH=4; blue), near neutral (pH=8; red) and alkaline (pH=12; green) pH simulations are represented. A sliding window average of 20 ns was applied to remove the undesired fast fluctuations.

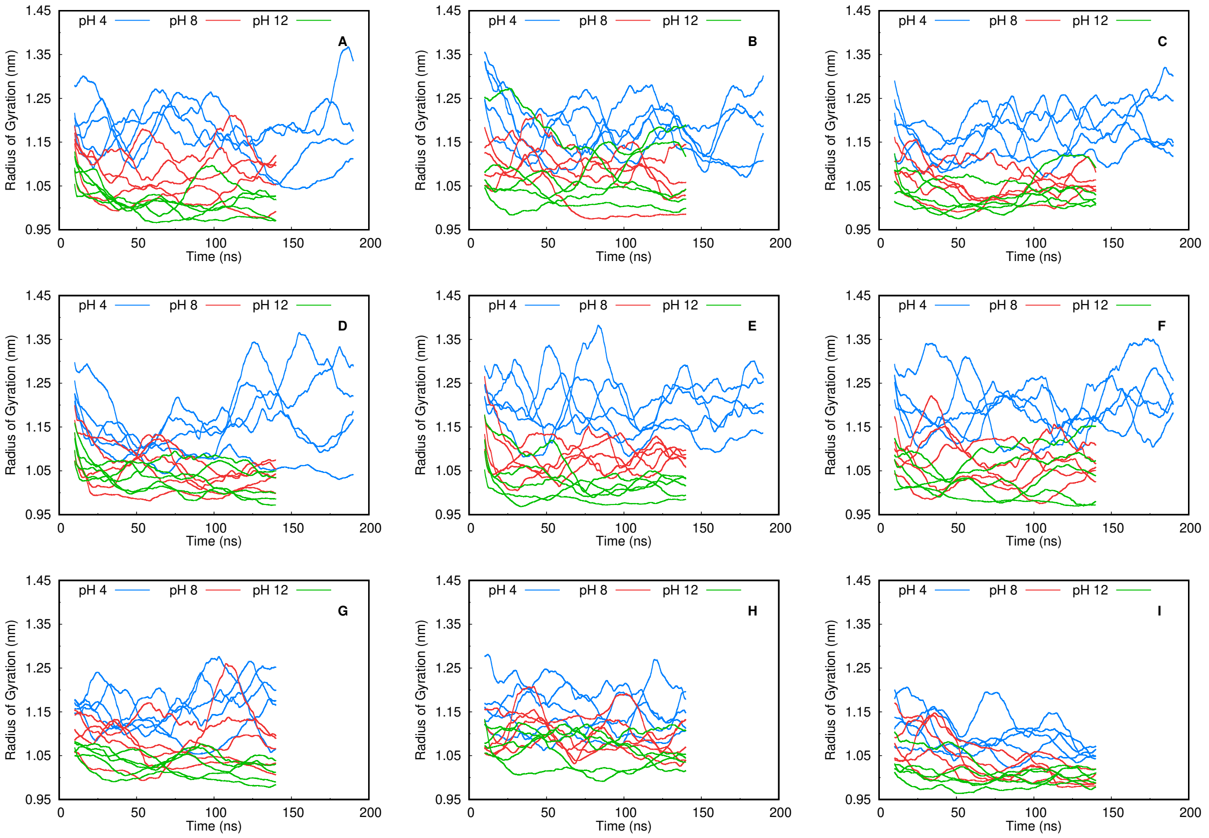

Figure S3: Radius of gyration of all systems, namely MH18 (A), DMH18 (B), MH18D1 (C), MH18D2 (D), MH18D3 (E), MH18D4 (F), MH13 (G), DMH13 (H) and MH47 (I) in water and over time. Five replicates of acidic (pH=4; blue), near neutral (pH=8; red) and alkaline (pH=12; green) pH simulations are represented. A sliding window average of 20 ns was applied to remove the undesired fast fluctuations.

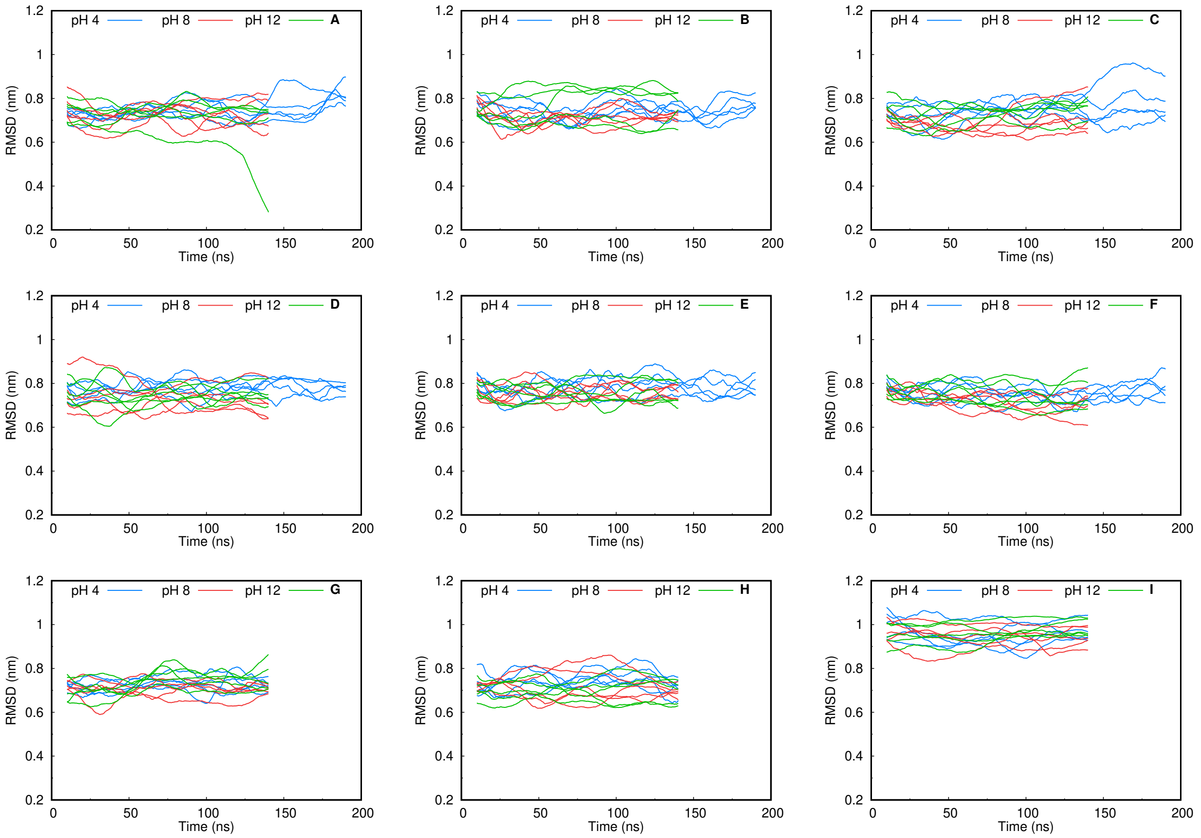

Figure S4: RMSD of all systems, namely MH18 (A), DMH18 (B), MH18D1 (C), MH18D2 (D), MH18D3 (E), MH18D4 (F), MH13 (G), DMH13 (H) and MH47 (I) in water and over time. Five replicates of acidic (pH=4; blue), near neutral (pH=8; red) and alkaline (pH=12; green) pH simulations are represented. One of the replicates for pH 12 of MH18 tends to 0 at the end of the simulation due to the last frame of this simulation being the central structure amongst all final frames. A sliding window average of 20 ns was applied to remove the undesired fast fluctuations.

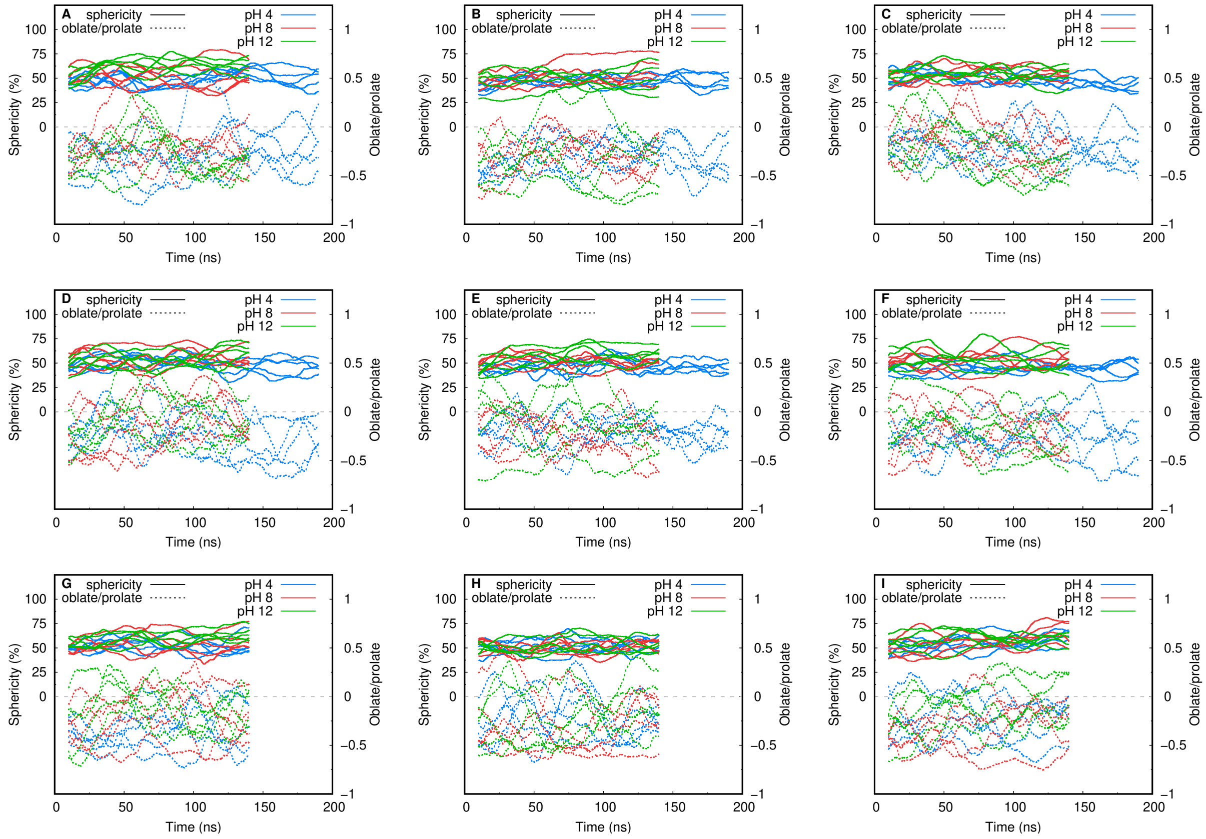

Figure S5: Sphericity of all systems, namely MH18 (A), DMH18 (B), MH18D1 (C), MH18D2 (D), MH18D3 (E), MH18D4 (F), MH13 (G), DMH13 (H) and MH47 (I) in water and over time. Five replicates of acidic (pH=4; blue), near neutral (pH=8; red) and alkaline (pH=12; green) pH simulations are represented. For the similarity to an oblate or a prolate, a positive percentage translates into a shape more oblate-like, while a negative percentage translates into a more prolate-like shape. A sliding window average of 20 ns was applied to remove the undesired fast fluctuations.

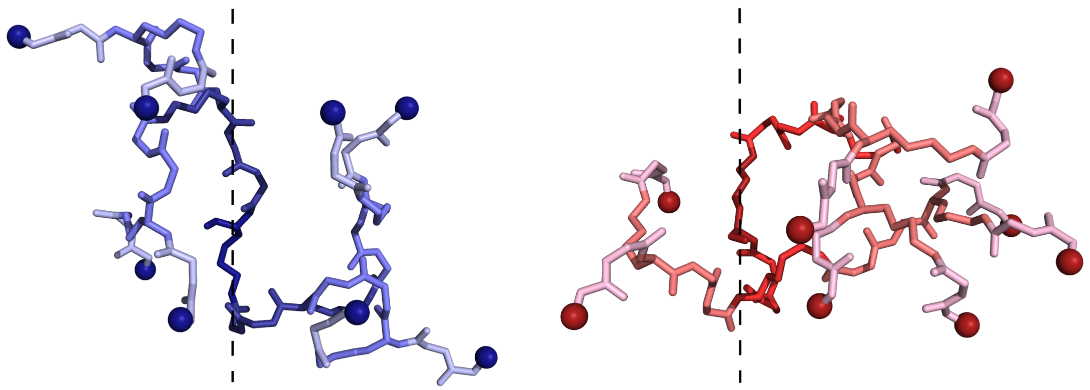

Figure S6: Representative conformations of the DMH18 dendrimer, at pH 12, on the low RMSD basin (left) and high RMSD basin (right) of its energy landscape (Figure 4I). The generations are shown with a gradient of blue and red, respectively, with the outermost generations having lighter colors. The main chains are depicted with sticks, and the nitrogen atoms from the N-termini are represented with spheres. Dashed lines are shown in both structures to mark the grouping of the branches from G2. The side chains and the G0 cores were omitted for clarity.

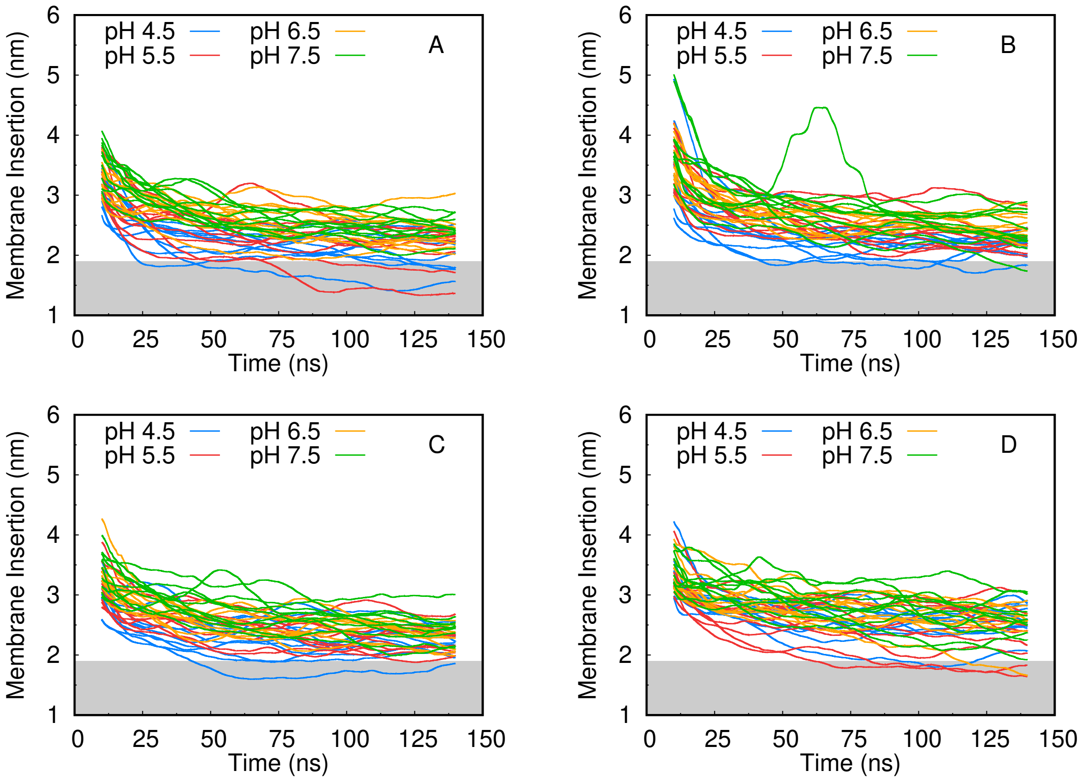

Figure S7: Distance to the center of the membrane for MH18 (A), MH18D3 (B), MH13 (C), and MH47 (D) over time. Ten replicates of pH 4.5, 5.5, 6.5, and 7.5 simulations are represented. A sliding window average of 20 ns was applied to remove the undesired fast fluctuations.

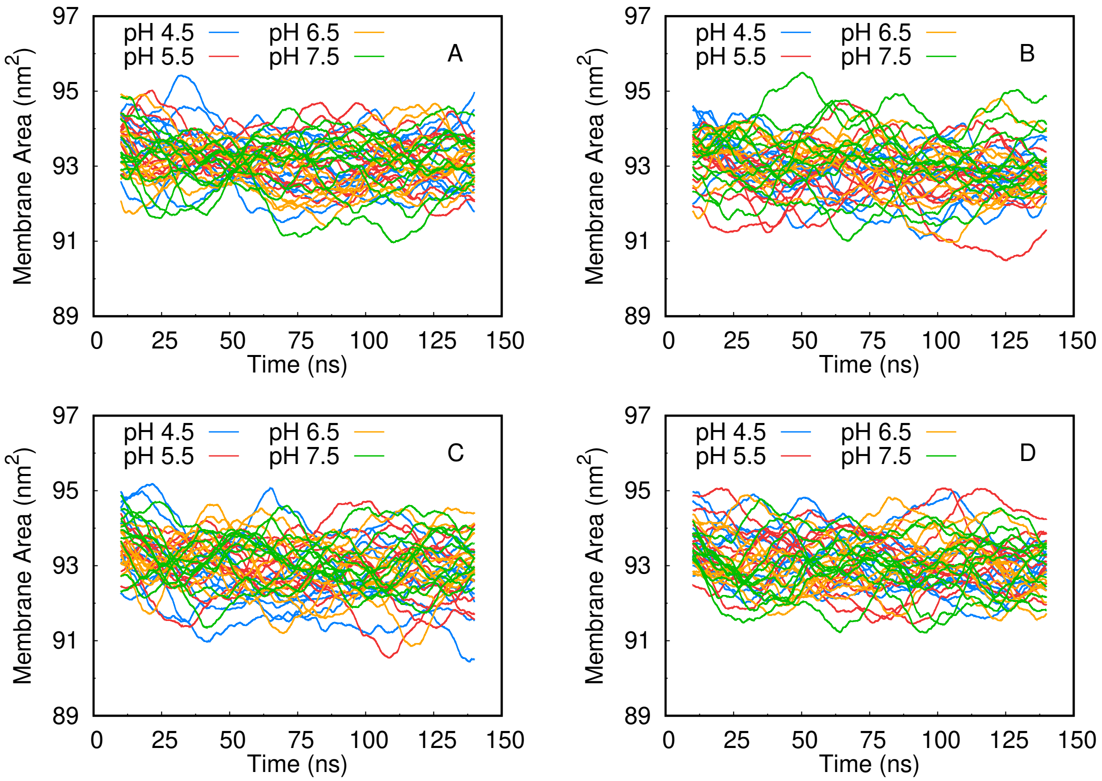

Figure S8: Membrane area of MH18 (A), MH18D3 (B), MH13 (C), and MH47 (D) over time. Ten replicates of pH 4.5, 5.5, 6.5, and 7.5 simulations are represented. A sliding window average of 20 ns was applied to remove the undesired fast fluctuations.

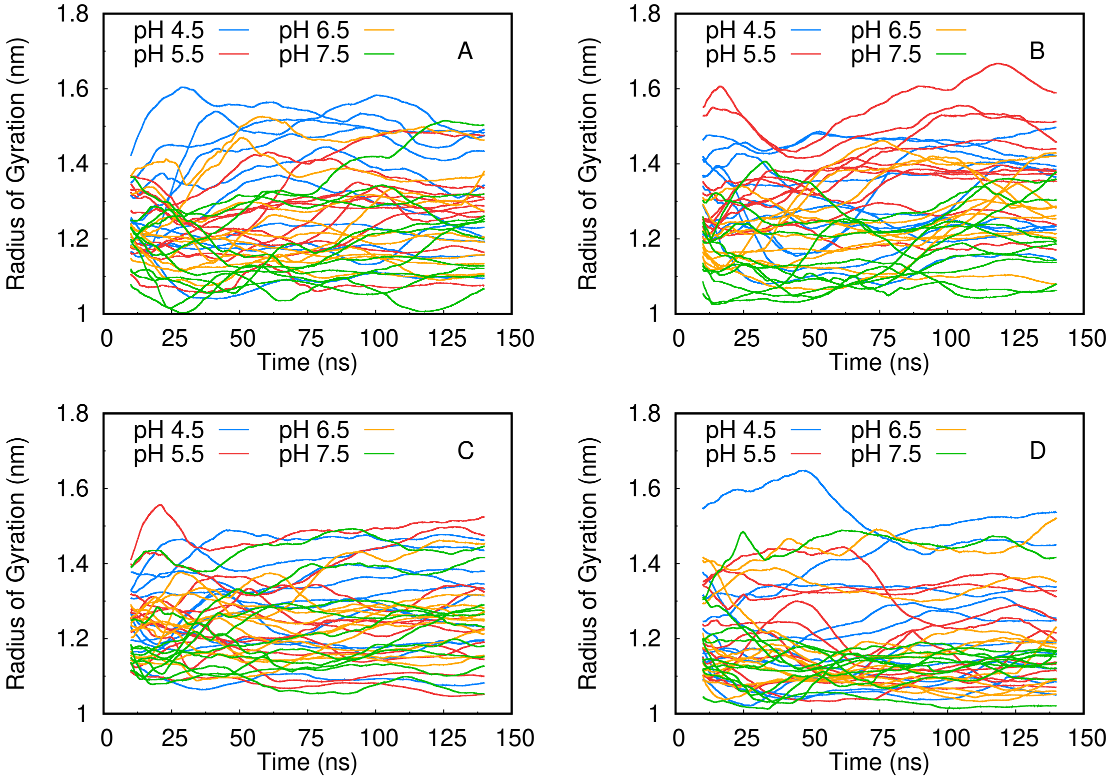

Figure S9: Radius of gyration of MH18 (A), MH18D3 (B), MH13 (C), and MH47 (D) over time. Ten replicates of pH 4.5, 5.5, 6.5, and 7.5 simulations are represented. A sliding window average of 20 ns was applied to remove the undesired fast fluctuations.

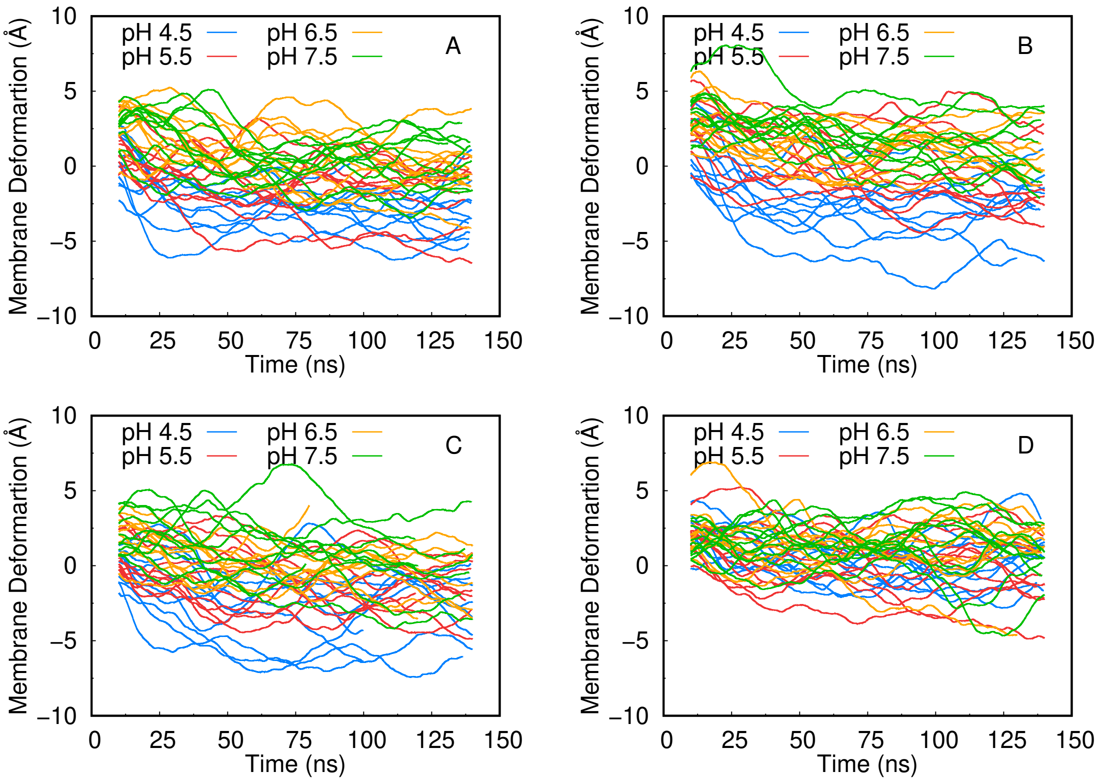

Figure S10: Local membrane deformation of MH18 (A), MH18D3 (B), MH13 (C), and MH47 (D) over time. Ten replicates of pH 4.5, 5.5, 6.5, and 7.5 simulations are represented. A sliding window average of 20 ns was applied to remove the undesired fast fluctuations.

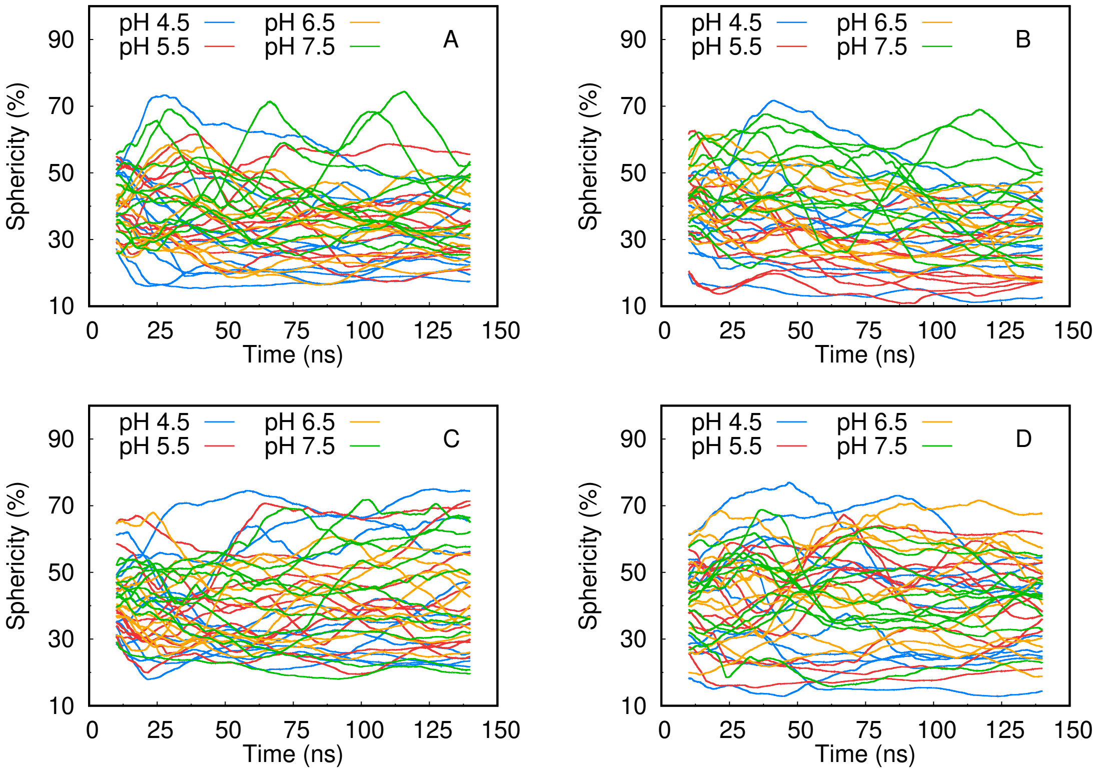

Figure S11: Sphericity percentage of MH18 (A), MH18D3 (B), MH13 (C), and MH47 (D) over time. Ten replicates of pH 4.5, 5.5, 6.5, and 7.5 simulations are represented. A sliding window average of 20 ns was applied to remove the undesired fast fluctuations.

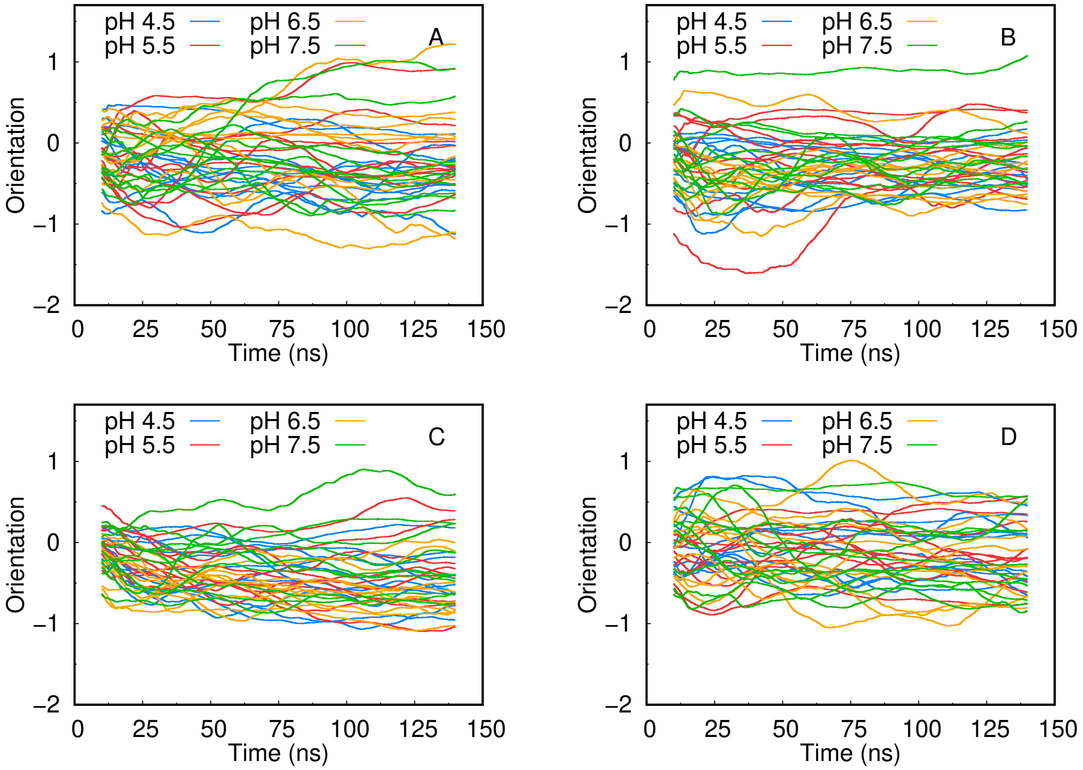

Figure S12: Orientation of MH18 (A), MH18D3 (B), MH13 (C), and MH47 (D) over time. Positive values represent the hydrophobic core (G0) of the dendrimer turned towards the membrane, while negative values represent the core pointing towards the water phase. Ten replicates of pH 4.5, 5.5, 6.5, and 7.5 simulations are represented. A sliding window average of 20 ns was applied to remove the undesired fast fluctuations.

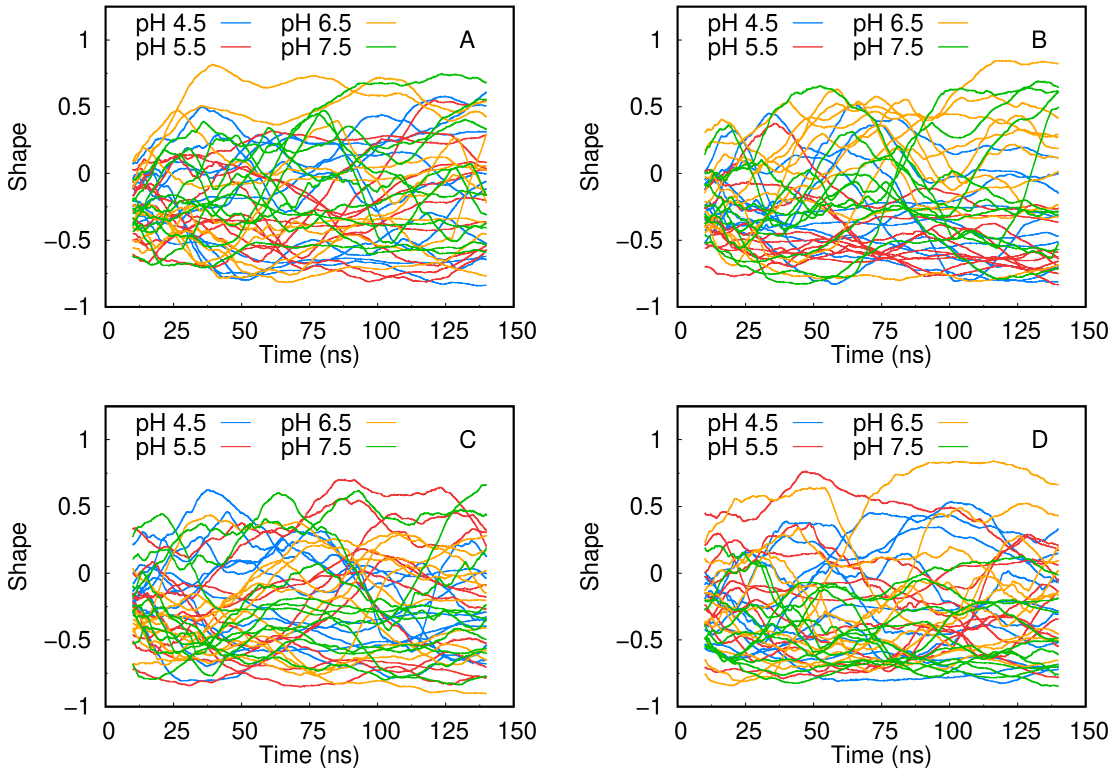

Figure S13: Shape of MH18 (A), MH18D3 (B), MH13 (C), and MH47 (D) over time. A positive percentage indicates how closely the dendrimer resembles an oblate or a disk, while a negative percentage indicates how closely it resembles a prolate or a cylinder. Ten replicates of pH 4.5, 5.5, 6.5, and 7.5 simulations are represented. A sliding window average of 20 ns was applied to remove the undesired fast fluctuations.

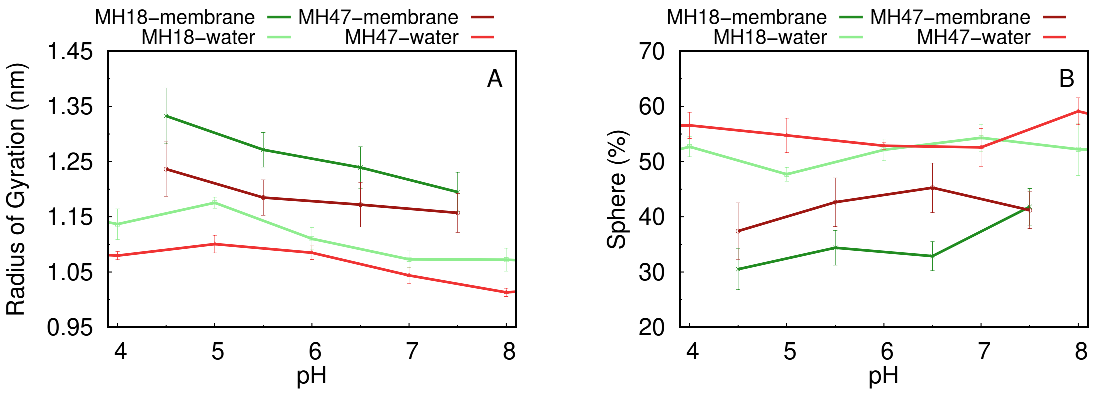

Figure S14: Radius of gyration (A) and average sphericity (B) for MH18 and MH47 in water and membrane simulations. MH18 and MH47 are shown in green and red, respectively. The water simulation data are in lighter tones, while the membrane ones are in darker colors.

Table S1: Percentage of PB/MC steps where at least one protonation change occurred, for all systems and all pH values in water.

pH	MH18	MH18D1	MH18D2	MH18D3	MH18D4	DMH18	MH13	DMH13	MH47
3	0.6 ± 0.1	0.6 ± 0.1	0.5 ± 0.0	0.5 ± 0.1	0.4 ± 0.0	0.8 ± 0.1	0.9 ± 0.2	0.7 ± 0.0	0.1 ± 0.0
4	5.5 ± 0.7	5.6 ± 0.4	5.2 ± 0.2	4.0 ± 0.4	4.6 ± 0.3	5.8 ± 0.5	5.9 ± 0.4	6.7 ± 0.8	2.5 ± 0.4
5	26.3 ± 1.4	26.8 ± 0.7	30.0 ± 0.9	26.0 ± 0.5	25.7 ± 0.9	28.4 ± 0.4	30.5 ± 0.8	30.4 ± 1.3	28.3 ± 0.6
6	57.6 ± 0.8	58.0 ± 0.3	58.6 ± 0.4	58.3 ± 0.8	58.2 ± 0.3	58.1 ± 0.6	57.4 ± 0.6	57.2 ± 0.6	57.3 ± 0.7
7	53.2 ± 1.1	50.1 ± 1.5	51.6 ± 1.0	53.1 ± 1.3	53.0 ± 1.5	53.9 ± 1.4	46.6 ± 2.8	47.1 ± 0.8	59.8 ± 1.3
8	33.7 ± 0.5	32.1 ± 0.3	33.2 ± 0.3	33.3 ± 0.5	32.2 ± 0.8	33.2 ± 0.3	32.5 ± 0.9	32.0 ± 0.4	26.2 ± 0.8
9	66.0 ± 0.5	66.5 ± 0.2	66.7 ± 0.7	66.8 ± 0.4	66.7 ± 0.3	66.3 ± 0.2	67.1 ± 0.2	66.5 ± 0.4	51.6 ± 0.9
10	78.0 ± 0.5	78.6 ± 0.2	78.6 ± 0.3	78.3 ± 0.3	78.4 ± 0.3	78.5 ± 0.4	78.1 ± 0.2	78.3 ± 0.4	75.1 ± 0.3
11	74.1 ± 0.4	74.3 ± 0.5	73.7 ± 0.5	73.5 ± 0.4	73.8 ± 0.4	74.4 ± 0.3	74.0 ± 0.3	74.2 ± 0.2	70.7 ± 0.2
12	32.6 ± 0.4	33.6 ± 0.5	32.5 ± 0.7	32.1 ± 0.3	33.5 ± 0.4	34.3 ± 0.6	32.6 ± 0.6	33.2 ± 0.8	28.0 ± 0.4
